# New benzimidazole-alkanesulfonate conjugates as cholinesterase inhibitors with in vitro and in silico validation

**DOI:** 10.1038/s41598-026-39534-z

**Published:** 2026-03-12

**Authors:** Mohamed A. Omar, Aisha A. K. Al-Ashmawy, Hayam A. Abd El Salam, Riham A. El-Shiekh, Wael Mahmoud Aboulthana, Aladdin M. Srour

**Affiliations:** 1 Chemistry of Natural and Microbial Products Department, Pharmaceutical and Drug Industries Research Institute, National Research Centre, Dokki, Giza, 12622 Egypt; 2 Department of Therapeutic Chemistry, National Research Centre, Dokki, Giza, 12622 Egypt; 3 Green Chemistry Department, Chemical Industries Research Institute, National Research Centre, Dokki, Giza, 12622 Egypt; 4 Department of Pharmacognosy, Faculty of Pharmacy, Cairo University, Kasr el Aini St., Cairo, 11562 Egypt; 5 Biochemistry Department, Biotechnology Research Institute, National Research Centre, 33 El Bohouth St., P.O. 12622, Dokki, Cairo Egypt

**Keywords:** Alzheimer, Benzimidazole, Alkanesulfonate, Acetylcholinesterase, Butyrylcholinesterase, Antioxidant, Biochemistry, Chemistry, Computational biology and bioinformatics, Drug discovery, Neuroscience

## Abstract

Alzheimer’s disease, the leading cause of dementia, is strongly associated with impaired cholinergic neurotransmission due to excessive acetylcholine degradation by acetylcholinesterase (AChE) and butyrylcholinesterase (BChE). Because current medications provide only symptomatic relief without modifying disease progression, there is an urgent need for more effective therapeutic agents. Dual inhibitors targeting both AChE and BChE represent a promising strategy. In this study, we designed and synthesized a novel series of benzimidazol-alkanesulfonate conjugates (**4a**–**r**) and evaluated their dual inhibitory activity against AChE and BChE. The results indicated that most compounds exhibited moderate to high inhibitory activity toward the target enzymes; however, derivatives **4b**, **4h**, **4i**, **4q**, and **4r** showed promising AChE inhibitory activity with IC_50_ values of 0.91 ± 0.02, 0.89 ± 0.02, 0.54 ± 0.05, 0.37 ± 0.01, and 0.41 ± 0.009 *µ*M, respectively, comparable to that of the reference drug donepezil (IC_50_ = 0.67 ± 0.00 *µ*M). The antioxidant potency (represented by TAC and IRP) and scavenging activity (represented by DPPH, ABTS, NO, OH, and H_2_O_2_) revealed that compounds **4q** and **4r** possess remarkable antioxidant potential, comparable to the reference antioxidant ascorbic acid. Furthermore, in silico ADME prediction and molecular docking studies were performed to predict their binding modes and interactions in the AChE binding pocket; the results revealed that derivatives **4q** and **4r** showed the highest binding affinity among the tested series and the reference drug donepezil, which validated the obtained in vitro results.

**Supplementary Information:**

The online version contains supplementary material available at 10.1038/s41598-026-39534-z.

## Introduction

Various environmental and genetic factors significantly influence the development of Alzheimer’s disease (AD). Despite extensive research, the exact cause and progression of AD are still not fully understood. The main pathological features include oxidative stress^1–3^, the formation of senile plaques from β-amyloid deposits^4^, neurofibrillary tangles caused by tau protein hyperphosphorylation^5^, and the selective loss of cholinergic neurons^6^. Strong evidence links acetylcholine (ACh) deficiency in the brains of AD patients to cognitive decline, especially memory problems^7^. Based on this, the most accepted treatment approach is based on the “cholinergic hypothesis,” which aims to improve cholinergic neurotransmission by inhibiting the enzymes acetylcholinesterase (AChE) and butyrylcholinesterase (BChE), responsible for breaking down ACh. A key structural difference between these enzymes is in their active sites: AChE contains aromatic residues like tryptophan and tyrosine that enable strong π–π interactions, while BChE replaces these with hydrophobic amino acids such as leucine and valine. This results in a wider and more flexible binding gorge, enabling accommodation of larger substrates^8,9^. Under normal conditions, AChE is the primary enzyme in the brain; however, in Alzheimer’s disease, BChE activity becomes increasingly important^10^. This shift emphasizes the urgent need for developing dual inhibitors that can target both AChE and BChE simultaneously. Current FDA (the U.S. Food and Drug Administration) approved medications for AD, including donepezil and galantamine (selective AChE inhibitors) and rivastigmine (a non-selective AChE inhibitor), only provide symptomatic relief and don’t stop or reverse disease progression^11^.

Benzimidazole is an aromatic heterocyclic compound formed by the fusion of a benzene ring with an imidazole ring. It is widely studied in pharmaceutical chemistry due to its versatile biological activities. Benzimidazole-containing compounds serve as core structures in many clinically important drugs. Its chemical stability, ability to form hydrogen bonds, and structural resemblance to purine bases contribute to its broad pharmacological relevance. Recent research highlights benzimidazole as a privileged scaffold in medicinal chemistry^12–14^.

**Fig. 1:**
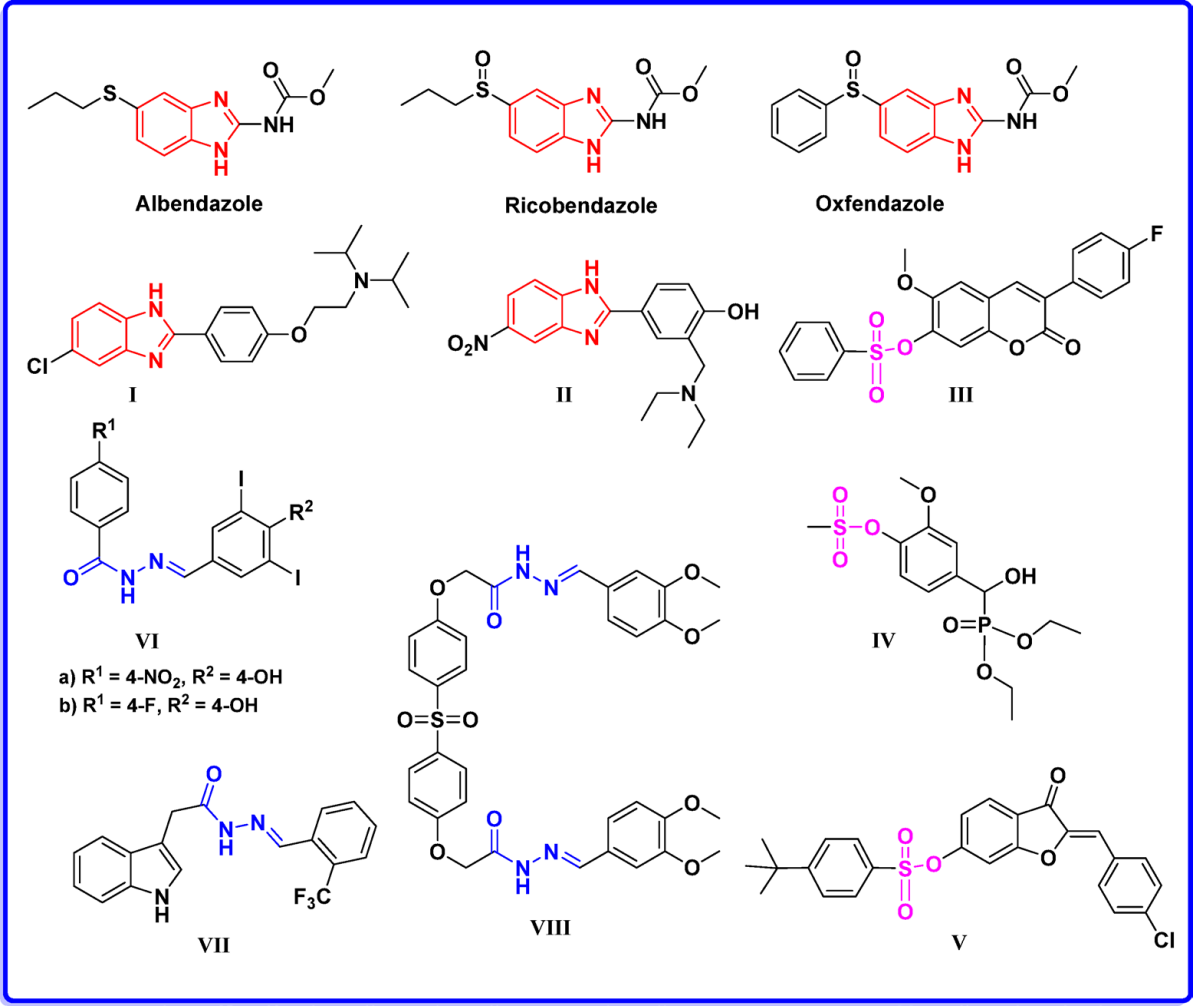
Some drugs and compounds containing benzimidazole, hydrazone, and sulfonate moieties as potent cholinesterase inhibitors.

The benzimidazole nucleus is a valuable heterocyclic scaffold in drug design. Additionally, some benzimidazole-based drugs, such as albendazole, ricobendazole, and oxbendazole, have shown promising inhibitory effects against AChE and BChE at the nanomolar level. Several studies have focused on the benzimidazole pharmacophore **I**–**II** in designing and developing notable cholinesterase inhibitors^15–19^, (Fig. 1). Moreover, Sulfonate-containing compounds have shown potential for suppressing cholinesterase enzymes (e.g., scopoletin-based arylsulfonate **III**)^20,21^. Furthermore, methanesulfonate derivative **IV** and aurone sulfonate derivative **V** have demonstrated promising AChE inhibitory activity (Fig. 1)^22,23^.

Compounds containing a hydrazide-hydrazone functional group are a significant class of pharmacologically effective chemical candidates that have attracted considerable interest among researchers. This moiety includes an azomethine group linked to a carbonyl group (-CONH-N=CH-), which is thought to exhibit various biological activities such as anti-inflammatory, antimicrobial, anticonvulsant, and anticancer effects^24–27^. Additionally, compounds **V**-**VII** have shown notable cholinesterase inhibitory effects, which play a role in breaking down the neurotransmitter ACh, a target for Alzheimer’s disease therapy (Fig. 1)^28–31^.

**Fig. 2:**
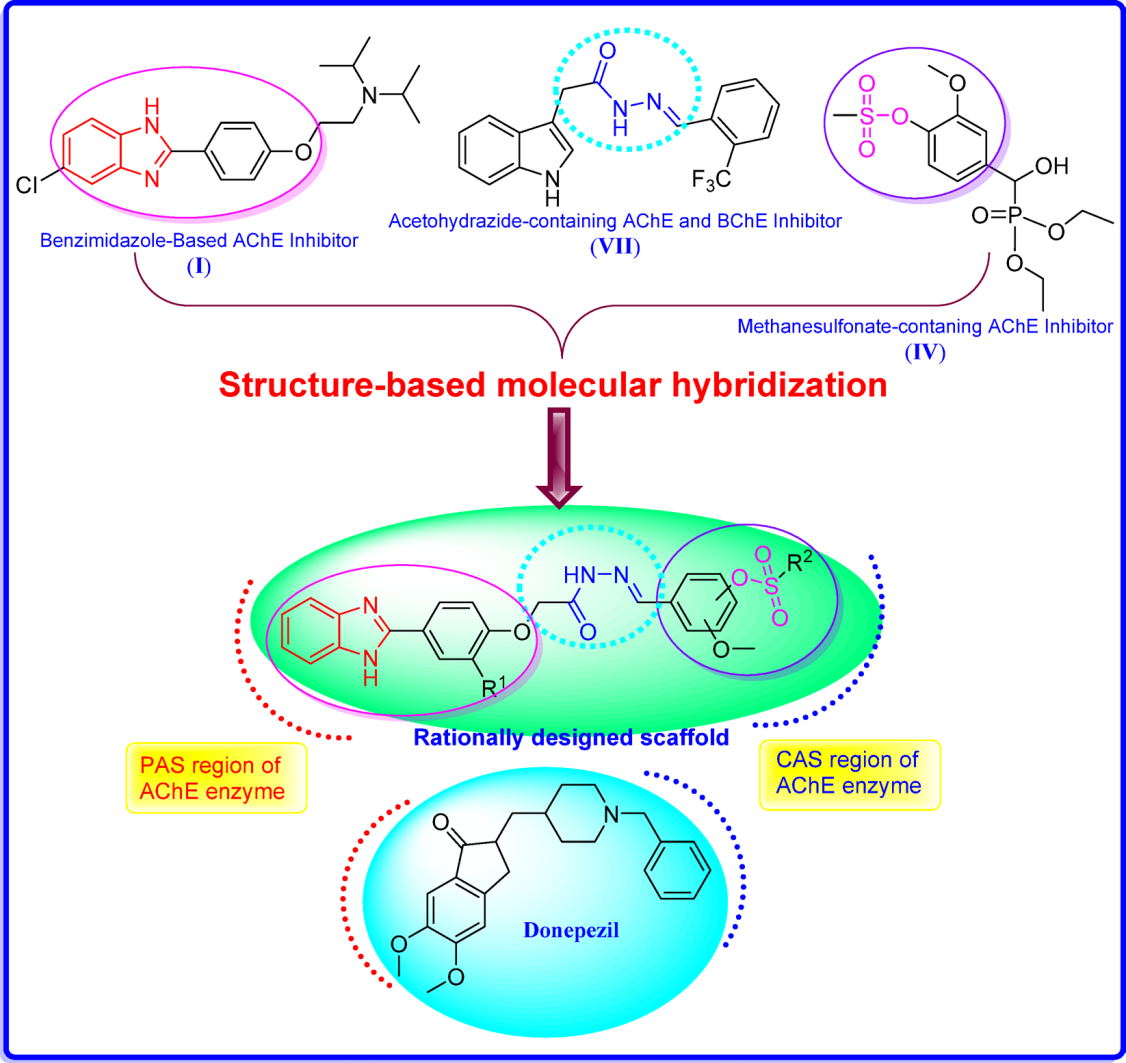
Rational design of benzimidazole-alkanesulfonate conjugates as promising cholinesterase inhibitors.

On the other hand, the donepezil molecule consists mainly of an aromatic system that can interact with the peripheral anionic site (PAS) and a basic center that can bind to the catalytic active site (CAS) of the AChE enzyme^32–34^. Thus, using the molecular hybridization technique, a novel series of the 2-phenylbenzimidazole-hydrazono-phenylalkane sulfonate conjugates was designed, synthesized, and evaluated for in vitro AChE and BChE inhibitory activity. The study was strengthened with in silico ADME (absorption, distribution, metabolism, and excretion) prediction and molecular docking for the promising conjugates in the AChE binding pocket, in which the phenylbenzimidazole moiety interacted with the PAS region and the phenylalkane sulfonate interacted with the CAS region, as shown in Fig. 2.

## Results and discussion

### Chemistry

**Scheme 1:**
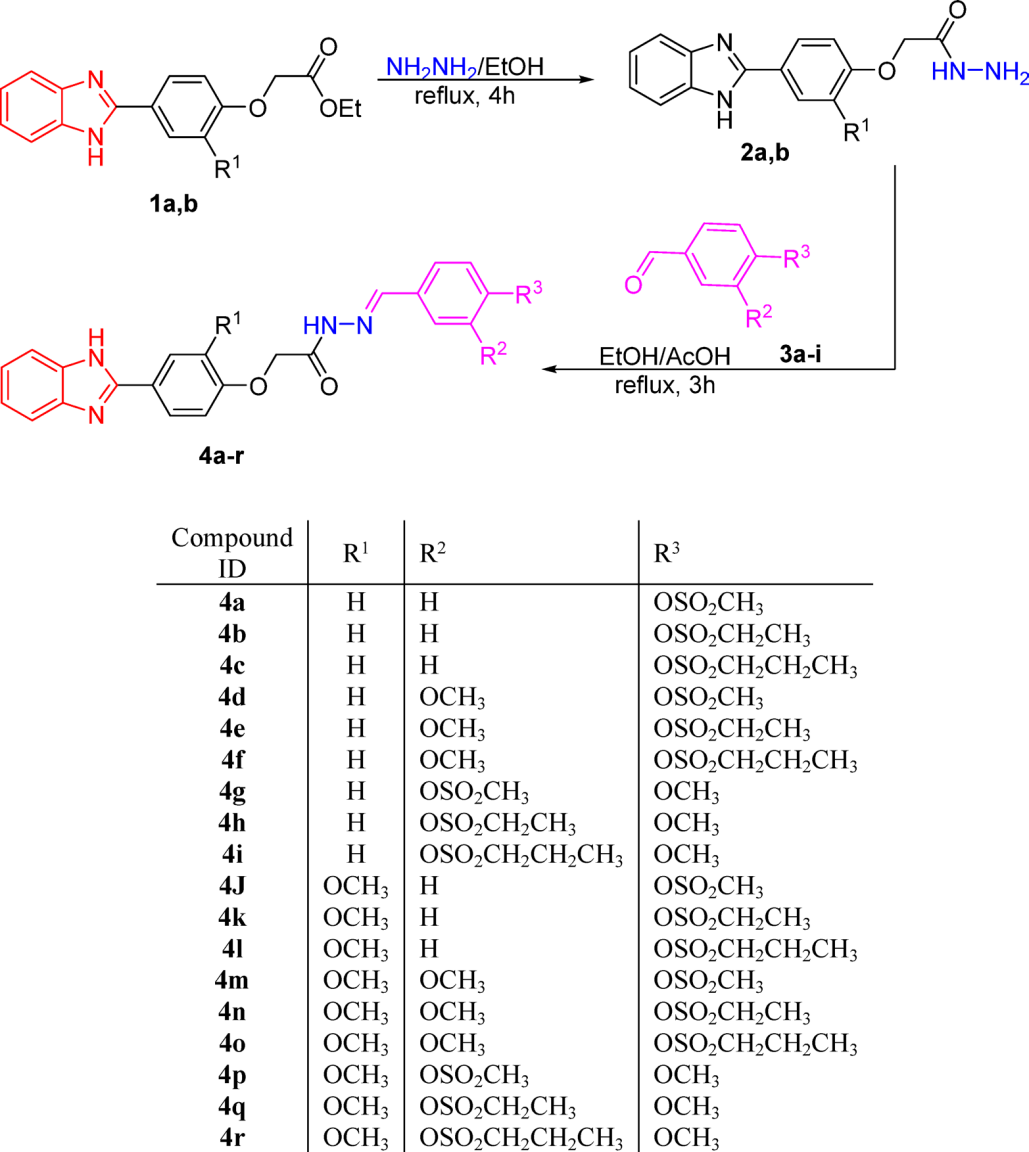
The synthetic pathway for synthesizing compounds **4a-r**.

The synthetic pathway for a novel series of benzimidazolyl phenoxy acetyl hydrazono alkanesulfonates (**4a-r**) depicted in Scheme 1 was initiated by heating *ortho*-phenylenediamine with ethyl 2-(4-formyl-2-methoxyphenoxy)acetate in the presence of a catalytic amount of sodium metabisulfite (Na_2_S_2_O_5_) in dimethyl formamide (DMF) for approximately 1 h. to afford 2-benzimidazolyl butanone (**1a**,**b**) which were then treated with hydrazine hydrate in ethanol, yielding the corresponding acetohydrazide derivatives (**2a**,**b**)^35^. These intermediates were subsequently allowed to react with a series of alkanesulfonyl aryl aldehydes (**3a**–**i**) under reflux conditions. The resulting precipitates were collected, dried, and recrystallized from appropriate solvents to afford the desired compounds (**4a-r**) as colorless microcrystals.

The chemical structures of the newly synthesized compounds **4a-r** were confirmed using IR, ^1^H and ^13^C NMR, spectroscopy, mass spectrometry, and elemental analysis. The IR spectra of compounds **4a-r** showed a broad absorption band at 3310–3327 cm^−1^ attributable to the hydroxyl group of the enolic (iminol) tautomer, together with bands at 3167–3189 cm^−1^ corresponding to the NH stretching vibration of the amide form. Additional characteristic absorptions confirmed the presence of C=O, C=N, and SO_2_ functionalities.

The ^1^H NMR spectra indicated that the hydrazone moiety exists in solution as an equilibrium mixture of iminol-amide (keto-enol-like) tautomers, arising from proton transfer between the hydrazide nitrogen and the carbonyl oxygen. This tautomeric behavior is evidenced by the presence of two distinct singlet signals (each integrating for 2 H) assigned to the OCH_2_C=O methylene protons, appearing at δ_H_ = 4.75–4.90 ppm for the iminol form and at δ_H_ = 5.25–5.37 ppm for the amide form. Similarly, two singlet signals integrating for one proton were observed for the azomethine (HC=N) proton at δ_H_ = 7.98–8.09 ppm and δ_H_ = 8.26–8.75 ppm, further confirming the coexistence of both tautomeric species. The alkyl sulfonate side chains were clearly identified by characteristic singlet, triplet-quartet, or triplet-sextet-triplet patterns in the aliphatic region, corresponding to methyl, ethyl, and propyl substituents, respectively. The ^13^C NMR spectra of compounds **4a-r** supported the proposed tautomeric equilibrium. Signals corresponding to alkanesulfonate carbons appeared in the upfield aliphatic region (δ_C_ = 8.07–55.81 ppm). The methylene carbon adjacent to the carbonyl group appeared as two distinct resonances at δ_C_ = 65.06–66.41 ppm (iminol form) and δ_C_ = 66.22–67.43 ppm (amide form). In addition, the azomethine carbon (C=N) resonated at δ_C_ = 142.51–146.50 ppm, while the amide carbonyl carbon (CONH) was observed in the range δ_C_ = 164.00-172.12 ppm (Figs. S1–S36).

This iminol-amide tautomerism is chemically significant, as it reflects enhanced conjugation within the hydrazone system and stabilization through intramolecular hydrogen bonding, which can influence molecular conformation, electronic distribution, and potentially the biological activity of these compounds.

### Anti-cholinesterase activity

The newly synthesized benzimidazole-alkanesulfonate conjugates **4a-r** were initially evaluated for percentage inhibition at 62.5 *µ*g/mL against both AChE and BChE, following the standard procedure^36–38^, as shown in Table 1. Most compounds exhibited moderate to high inhibitory activity toward the target enzymes. However, only five derivatives, **4b**, **4h**, **4i**, **4q**, and **4r**, showed more than 60% inhibition. Specifically, these compounds displayed AChE inhibition values of 65.40%, 60.74%, 69.64%, 74.02%, and 70.99%, respectively. In contrast, compounds **4d**, **4e**, **4f**, **4i**, and **4l ** showed moderate activity against BChE with inhibition rates ≥ 50%; none achieved significant inhibition (i.e., exceeding 60%).

**Table 1:** % inhibition of the newly synthesized conjugates (**4a-r**) against AChE and BChE.

Compd. No.	Percentage inhibition at 62.5 *µ*g/ml	
	AChE	BChE
**4a**	40.87	35.08
**4b**	65.40	36.77
**4c**	40.18	33.87
**4d**	44.72	49.76
**4e**	38.96	50.35
**4f**	44.24	50.10
**4g**	49.32	41.59
**4h**	60.74	38.56
**4i**	69.64	49.86
**4j**	48.43	48.75
**4k**	45.88	44.93
**4l**	44.07	50.69
**4m**	43.03	45.28
**4n**	48.19	44.85
**4o**	40.94	36.66
**4p**	45.84	37.53
**4q**	74.02	33.60
**4r**	70.99	32.51

Accordingly, the top five active derivatives were subjected to dose-dependent screening within a concentration range of 125−1.95 *µ*g/mL to determine their IC_50_ values against AChE. The obtained IC_50_ values were 0.91 ± 0.02, 0.89 ± 0.02, 0.54 ± 0.05, 0.37 ± 0.01, and 0.41 ± 0.009 *µ*M for **4b**, **4h**, **4i**,** 4q**, and **4r**, respectively, comparable to that of the reference drug donepezil (IC_50_ = 0.67 ± 0.00 *µ*M), Table 2.

The data obtained revealed that derivative **4q** was the most potent among the tested compounds. The activity order for the other compounds was as follows: **4r** > **4i** > **4h** > **4b**. This confirms that the presence of two methoxy groups within the benzimidazole-alkanesulfonate conjugate scaffold enhances AChE inhibitory activity, as demonstrated by compounds **4q** and **4r**. In contrast, compounds with only one methoxy group (**4h** and **4i**) exhibited lower activity, while the derivative lacking any methoxy groups (**4b**) showed the least potency. These findings suggest that these conjugates could serve as a foundational nucleus for the development of more potent anti-acetylcholinesterase agents.

**Table 2:** IC_50_ values of the best new conjugates **4b**, **4h**, **4i**,** 4q**, and **4r** against AChE.

Compd. No.	Acetylcholine esterase
	IC_50_ (Average ± SD)^a^ (*µ*M)
**4b**	0.91 ± 0.02
**4h**	0.89 ± 0.02
**4i**	0.54 ± 0.05
**4q**	0.37 ± 0.01
**4r**	0.41 ± 0.009
**Donepezil**	0.67 ± 00

^a^SD Standard deviation.

### Antioxidant activity

Acetylcholinesterase (AChE) and butyrylcholinesterase (BChE) not only regulate cholinergic neurotransmission but also modulate oxidative stress through direct interactions with reactive oxygen species (ROS). Both enzymes possess free radical scavenging properties that help protect neurons from oxidative damage, a process closely linked to Alzheimer’s disease (AD), where oxidative stress and cholinergic dysfunction coexist. Since AChE inhibitors, the main therapeutic approach for AD, can also affect BChE activity, they may indirectly influence the brain’s antioxidant balance. The functional interplay between these enzymes may further modulate neuroinflammation, suggesting that targeting their dual roles could offer therapeutic benefits by enhancing antioxidant defenses while restoring cholinergic function in the aging brain. Free radicals are highly reactive species that contain unpaired electrons, causing oxidative stress and cellular damage when uncontrolled. Antioxidants counteract these effects by neutralizing reactive oxygen and nitrogen species (ROS and RNS), including O_2_^−^, H_2_O_2_, OH^−^, and NO_2_^+^, which are implicated in various pathological conditions such as inflammation, neurodegenerative diseases, ischemia/reperfusion injury, and cancer^39–41^.

The antioxidant properties of the newly synthesized benzimidazole-alkanesulfonate conjugates (**4b**, **4h**, **4i**,** 4q**, and **4r**) were evaluated using several in vitro assays, including total antioxidant capacity (TAC), iron regulatory protein (IRP) levels, and radical scavenging activities DPPH (2,2-Diphenyl-1-picrylhydrazyl), ABTS (2,2′-Azino-bis(3-ethylbenzothiazoline-6-sulfonic acid)), NO (Nitric oxide), OH (Hydroxyl radical), and H_2_O_2_ (Hydrogen peroxide), Tables S1,S2.

Compound **4q** exhibited the highest total antioxidant capacity (83.05 ± 0.98 mg gallic acid/g) and the highest IRP level (123.4 ± 1.45 *µ*M), which are close to those of the standard ascorbic acid (99.43 ± 1.16 mg gallic acid/g and 451.6 ± 5.28 *µ*M, respectively), indicating a strong overall antioxidant potential. Derivative **4r** also showed notable TAC (66.80 ± 0.93 mg gallic acid/g) and IRP (96.7 ± 1.34 *µ*M) values, whereas **4h** recorded the lowest readings, with an IRP value of 29.0 ± 0.67 *µ*M. Among all tested compounds, **4q** displayed the lowest IC_50_ values of 11.38 ± 0.46 *µ*M (DPPH), 9.88 ± 0.20 *µ*M (ABTS), 14.45 ± 0.37 *µ*M (NO), 18.68 ± 0.48 *µ*M (OH), and 21.15 ± 0.58 *µ*M (H_2_O_2_), which are comparable to those of ascorbic acid (29.07 ± 0.51, 25.51 ± 0.45, 36.01 ± 0.62, 46.56 ± 0.34, and 52.76 ± 0.51 *µ*M, respectively). This indicates that compound **4q** possesses strong radical scavenging efficiency despite its higher molecular weight (Table 3). Compound **4r** ranked second, with IC_50_ values of 13.81 ± 0.58 *µ*M (DPPH), 11.80 ± 0.24 *µ*M (ABTS), 18.15 ± 0.45 *µ*M (NO), 23.49 ± 0.65 *µ*M (OH), and 26.60 ± 0.76 *µ*M (H_2_O_2_). Compounds **4b** and **4i** showed moderate activity, whereas 4 h exhibited the weakest antioxidant performance, with IC_50_ values exceeding 180 *µ*M for OH and H_2_O_2_ radicals, indicating poor scavenging capability.

The observed variation in antioxidant activity among the tested compounds may be attributed to the electronic nature of their substituents. The presence of electron-donating methoxy groups on the aromatic rings of derivatives **4q** and **4r** enhances electron density and facilitates hydrogen or electron transfer to reactive radicals, thereby stabilizing the resulting intermediates through resonance effects (Table 3).

**Table 3:** In vitro antioxidant and radical scavenging activities of compounds **4b**, **4h**, **4i**,** 4q**, and **4r** expressed as IC_50_ values (*µ*M) against DPPH, ABTS, NO, OH, and H_2_O_2_ radicals at equal concentrations (10 *µ*g/mL).

Sample ID	IC_50_ µM				
	DPPH	ABTS	NO	OH	H_2_O_2_
**4b**	20.17 ± 0.36	16.95 ± 0.31	27.96 ± 0.52	36.18 ± 0.29	40.96 ± 0.46
**4h**	54.23 ± 1.02	39.11 ± 0.55	142.45 ± 5.53	184.29 ± 7.20	208.64 ± 7.68
**4i**	25.51 ± 1.09	20.71 ± 0.44	39.09 ± 1.15	50.57 ± 1.63	57.26 ± 1.89
**4q**	11.38 ± 0.46	9.88 ± 0.20	14.45 ± 0.37	18.68 ± 0.48	21.15 ± 0.58
**4r**	13.81 ± 0.58	11.80 ± 0.24	18.15 ± 0.45	23.49 ± 0.65	26.60 ± 0.76

Values are expressed as mean ± standard deviation (*n* = 3).

## In Silico study

### In silico drug likeness and ADME prediction studies

**Fig. 3:**
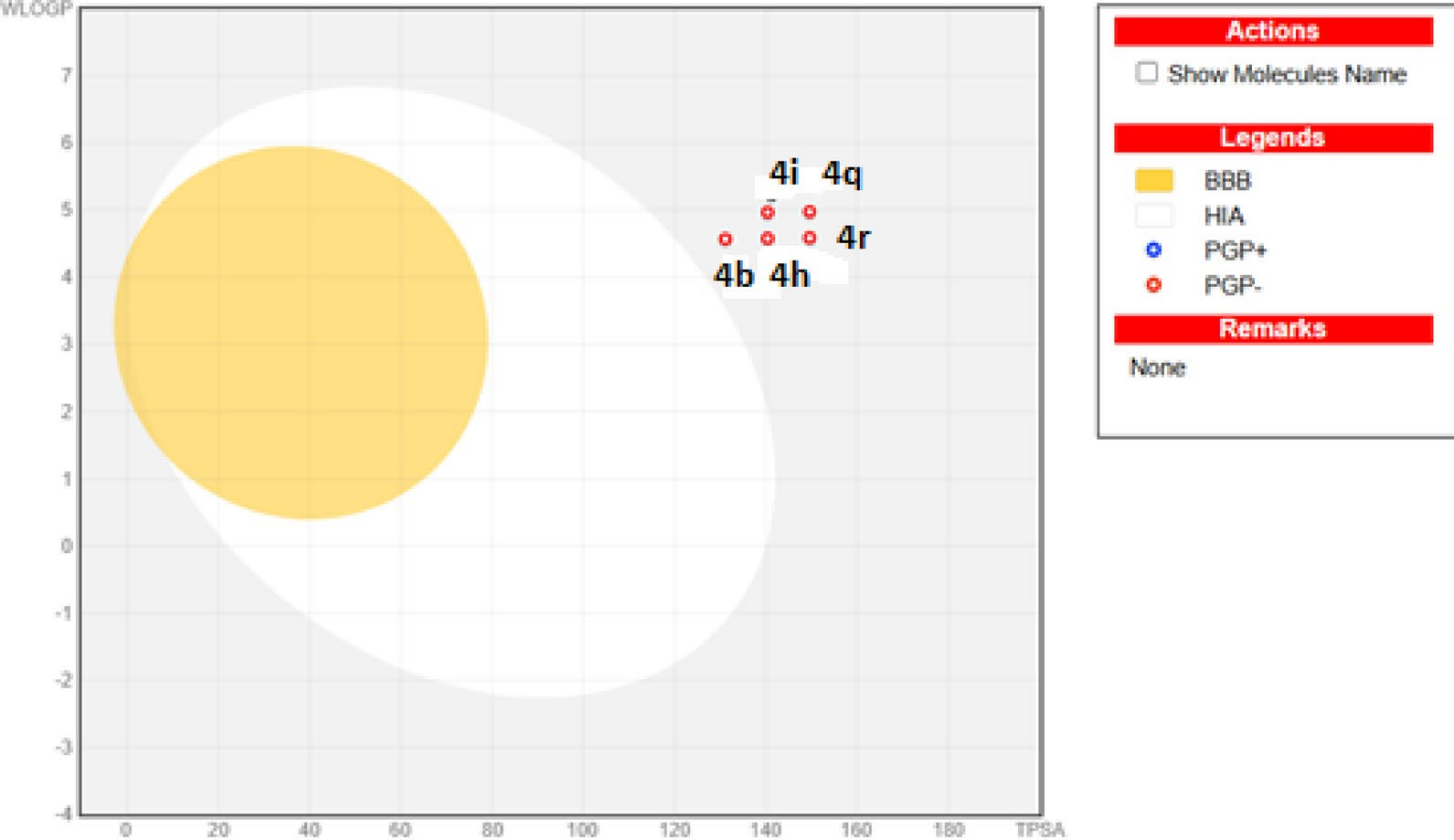
Boiled-Egg plot downloaded from the Swiss ADME website http://www.swissadme.ch for derivatives **4b**, **4i**, **4h**, **4q**, and **4r**.

Building upon the previously mentioned in vitro AChE inhibitory screening results, the five promising derivatives, namely **4b**, **4h**, **4i**, **4r**, and **4q**, were assessed for an in silico ADME (absorption, distribution, metabolism, and excretion) prediction study using the Swiss ADME website; http://www.swissadme.ch as demonstrated in Tables 4 and 5, and Fig. 3^42^.

In Table 4, it was shown that **4b** followed the Lipinski drug likeness rule without any violations. On the other side, one violation was Mwt > 500 in **4h** and **4i**, and two violations were Mwt > 500 and the number of rotatable bonds > 10 in **4q** and **4r**. For aqueous solubility, all the tested compounds were found to be poorly water-soluble. All of the tested derivatives were assumed to pass the Pan-assay interference (*PAINS*) filter, which examined the presence of substructures that may interfere with any biochemical activity. Concerning pharmacokinetics parameters prediction, the inhibition profile prediction of some representative of CYP450’s class family was tabulated in Table 5, in which these enzymes affects the metabolism and excretion of any drug, Also, the best five tested derivatives were expected to be of moderate skin permeability and of good oral bioavailability except **4q** and **4r** were presumed to be of very low oral bioavailability. As illustrated in Fig. 3, in the Boiled-Egg plot^43^, all five derivatives were predicted to have a low GIT absorption rate, could not cross the blood-brain barrier (BBB), and were not expected to be a substrate for the efflux transporter P-glycoprotein.

**Table 4:** Swiss ADME prediction displaying drug-likeness (Lipinski’s rule) and various physicochemical properties for derivatives **4b**, **4i**, **4h**, **4r**, and **4q**.

Sample ID	Lipiniski rules								Water solubility	Ali Class	PAINS
	MW	Heavy atoms	Rotatable bonds	HB acceptors	HB donors	MLOGP	TPSA	violations	Ali Log S		#alerts
**4b**	478.5	34	10	7	2	2.53	131.1	0	− 6.47	Poorly soluble	0
**4h**	508.6	36	11	8	2	2.23	140.4	1	− 6.63	Poorly soluble	0
**4i**	522.6	37	12	8	2	2.44	140.4	1	− 7.17	Poorly soluble	0
**4q**	538.6	38	12	9	2	1.94	149.6	2	− 6.79	Poorly soluble	0
**4r**	552.6	39	13	9	2	2.14	149.6	2	− 7.34	Poorly soluble	0

*MW: Molecular weight ≤ 500; Heavy atoms: 20 ≤ atoms ≤ 70; Rotatable bonds ≤ 9; lipophilicity: MlogP < 4.15; HB acceptor: Hydrogen bond acceptor ≤ 10; HB Donor: Hydrogen bond donor ≤ 5; TPSA (Topological polar surface area) 20–130 A^◦2^.

**Table 5:** The prediction of some Pharmacokinetic parameters, including the cytochrome P450 inhibitory profile, the skin permeability, and the oral bioavailability score.

Sample ID	CYP450 inhibitors					Skin permeability	Bioavailability score
	CYP1A2	CYP2C19	CYP2C9	CYP2D6	CYP3A4	log Kp (cm/s)	
**4b**	No	Yes	Yes	No	Yes	-6.37	0.55
**4h**	No	Yes	Yes	No	Yes	-6.58	0.55
**4i**	No	Yes	Yes	No	Yes	-6.29	0.55
**4q**	No	Yes	Yes	No	Yes	-6.78	0.17
**4r**	No	Yes	Yes	Yes	Yes	-6.49	0.17

*The higher the negative log Kp, the more the skin impermeability; the score of bioavailability ≥ 0.55 indicates a good oral bioavailable compound.

### Molecular docking of derivatives **4b**, **4i**, **4h**, **4r**, and **4q** in the ache active site

**Fig. 4:**
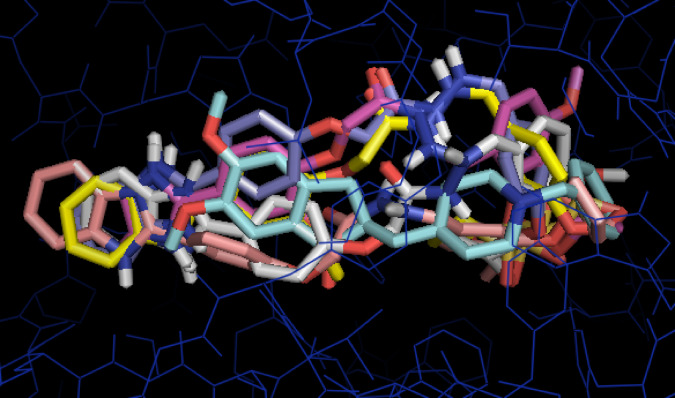
The predicted collective 3D shape of Donepezil (light blue) superimposed over the five tested compounds, **4b** (violet), **4h** (white), **4i** (light orange), **4q** (pink), and **4r** (yellow) in the AChE active site (PDB code: 4EY7) is represented in dark blue sticks. Generated by *Biovia Discovery Studio 2024* (https://discover.3ds.com/).

**Fig. 5:**
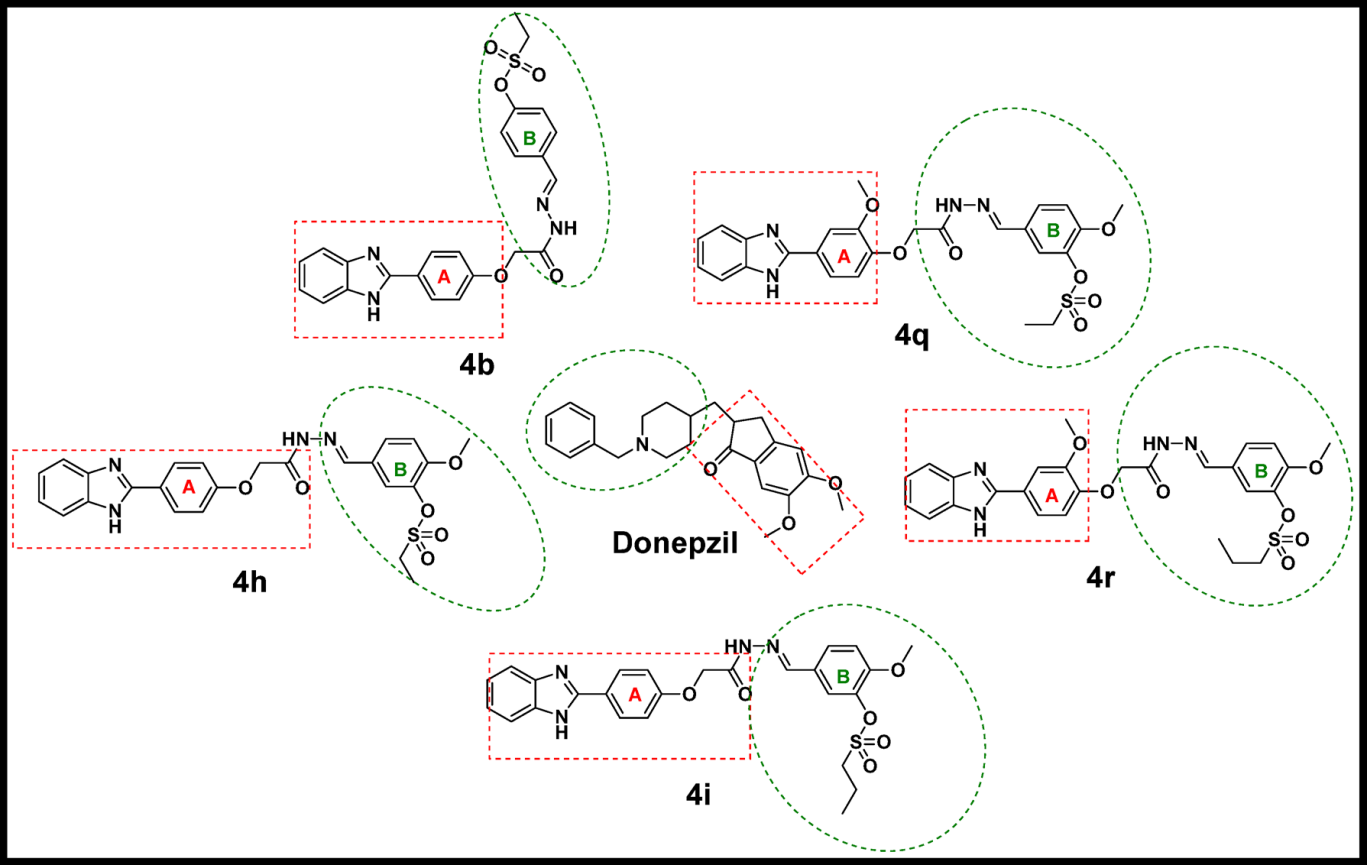
A representative structure-based figure illustrating the predicted binding pockets in AChE for Donepezil, **4b**, **4i**, **4h**, **4q**, and **4r** in which the peripheral anionic domain (PAS) was represented in a red dotted rectangle, while the catalytic active site (CAS) was shown in green dotted oval.

**Fig. 6:**
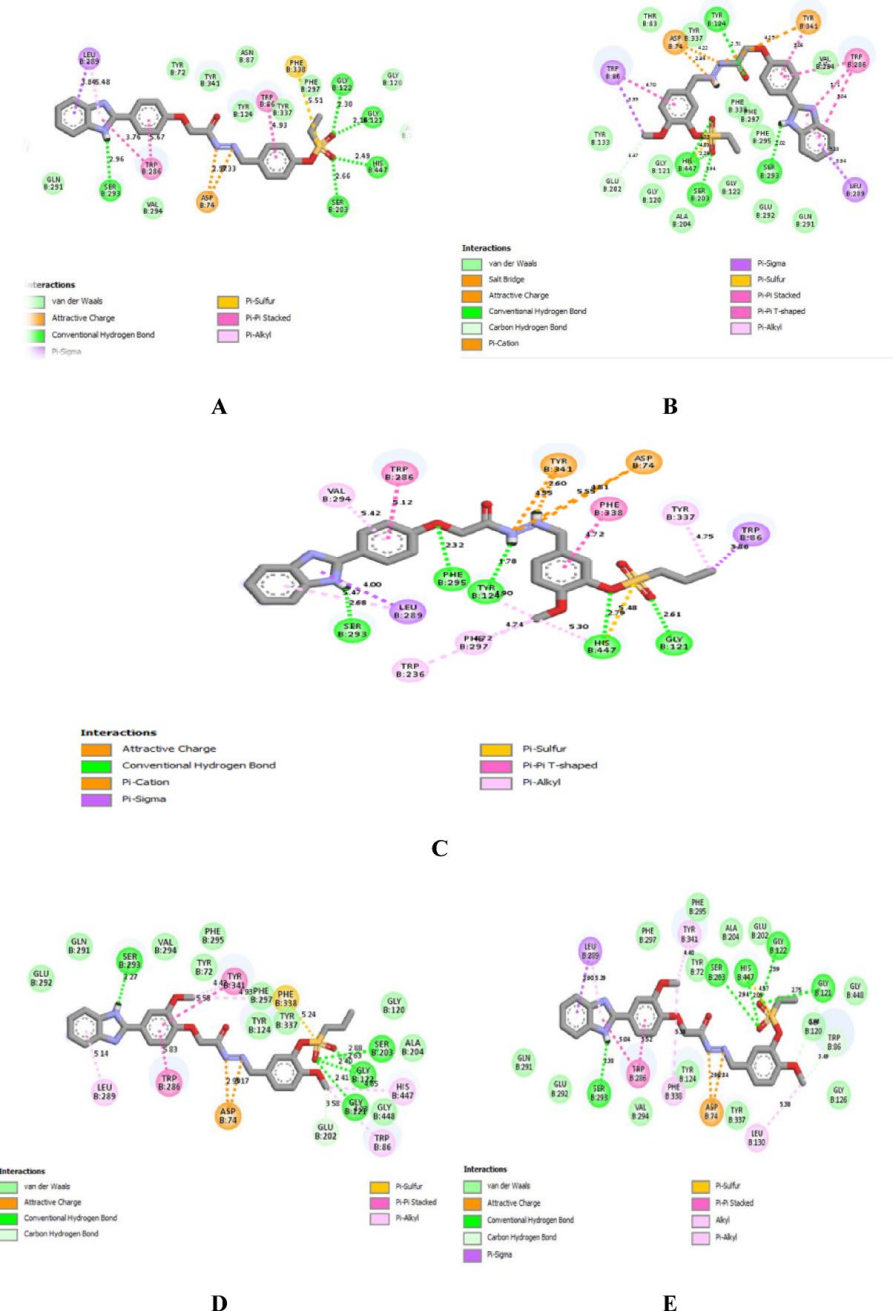
The predicted 2D-binding interactions of conjugates in the active site of AChE (PDB code: 4EY7) A: **4b**, B: **4h**, C: **4i**. D: **4q**, and E: **4r**. Generated by *Biovia Discovery Studio 2024* (https://discover.3ds.com/).

For a deeper explanation of the promising descending cholinesterase inhibitory activity of **4q**, **4r**, **4i**, **4h**, and **4b**, their predicted binding mode and interactions in the AChE binding pocket, the docking simulation was carried out utilizing the Autodock Vina wizard PyRx (https://pyrx.sourceforge.io/*)* as the previously published method^44–46^. The PDB code of the acetylcholine esterase ( PDB ID: 4EY7)^47^was downloaded from the Protein Data Bank (https://www.rcsb.org/) and underwent energy minimization using the YASARA energy minimization server^48^. For the validation of the docking procedure, the native co-crystallized ligand, donepezil, was redocked, and its RMDS was 0.5 A^◦^. *Biovia Discovery Studio 2024* (https://discover.3ds.com/*)* was used to demonstrate the 2D and 3D figures of the binding interactions, as well as the superimposition on the native ligand. The results were represented in Table 6; Figs. 4, 5 and 6. Interestingly, all five tested derivatives bound similarly and superimposed over the native ligand donepezil in the AChE binding pocket as presented in Fig. 4. Generally, the benzimidazole and the aryl ring A were predicted to occupy the peripheral anionic domain (PAS) while the aryl ring B with the alkyl sulfonate moiety mainly were expected to bound in the catalytic active site (CAS) imitating the previously reported binding mode of the benzylpiperidine motif and fused aromatic ring moiety of donepezil respectively, as sketched in Fig. 5^49^.

According to the binding score, the compounds were arranged in the descending affinity order as follows: **4r**, **4q**, **4h**, **4i**, donepezil, and **4b**, as shown in Table 6. The higher stability of our compounds might be attributed to the number of hydrogen bonds (4–5) they were predicted to form in the binding site, while donepezil formed only one hydrogen bond with Phe 295.

**Table 6:** The binding score (Kcal /mol) of **4b**, **4h**, **4i**, **4r**, and **4q**, in addition to the native ligand donepezil in ache (PDB code: 4EY7and detailed binding interactions and bond lengths.

Compound	Binding affinity (Kcal /mol)	Detailed binding types and bond lengths.
**4b**	− 10.5	*Hydrogen bond*: Gly 121 (2.15 A◦), Gly 122 (2.30 A◦), Ser 203 (2.66 A◦), His 447 (2.49 A◦); *Electrostatic attraction*: Asp 74 (2.33 A◦, 2.94 A◦); Pi- sulfur: Phe 338 (5.51 A◦); *Hydrophobic interactions*: Van der waals; Pi- alkyl: Leu 289 (5.48 A◦); Pi- pi: Trp 286 (3.76 A◦,5.67 A◦ ); Pi- sigma : Leu 289 (3.85 A◦).
**4h**	− 10.8	*Hydrogen bond*: Tyr 124 (2.61 A^◦^), His 447 (1.75 A^◦^, 2.29 A^◦^), Ser 203 (1.94 A^◦^), Ser 293 (2.02 A^◦^); *Carbon-hydrogen*: Glu 202 (3.47 A^◦^); *Electrostatic attraction*: Asp 74 (2.34 A^◦^, 4.22 A^◦^), Tyr 341(4.25 A^◦^); *Pi- sulfur*: His 447 (4.83 A^◦^); *Hydrophobic interactions*: Van der waals; Pi- alkyl: Leu 289 (5.88 A^◦^) ; *Pi- pi*: Trp 286 (5.17 A^◦^,5.71 A^◦^, 5.84 A^◦^ ), Tyr 341 (5.06 A^◦^), Trp 86 (4.70 A^◦^) ; Pi- sigma: Leu 289 (3.34 A^◦^), Trp 86 (3.99 A^◦^).
**4i**	− 11.0	*Hydrogen bond*: Gly 121 (2.61 A◦), His 447 (2.79 A◦), Try 124 (1.78 A◦), Phe 295 (2.32 A◦), Ser 293 (2.68 A◦); *Electrostatic attraction*: Asp 74 (4.81 A◦, 5.55 A◦), Tyr 341(4.55 A◦, 2.60 A◦); Pi- sulfur: His 447 (5.48 A◦); *Hydrophobic interactions*: Van der waals; Pi- alkyl: Trp 236 (4.74 A◦), His 447 (5.30 A◦), Val 294 (5.42 A◦), Phe 297(4.93 A◦), Tyr 337 (4.75 A◦); Pi- pi: Trp 286 (5.12 A◦), Phe 338 (4.72 A◦); Pi- sigma: Leu 289 (4.00 A◦), Trp 86 (3.86 A◦).
**4q**	− 11.5	*Hydrogen bond*: Gly 121 (2.75 A◦), Gly 122 (2.59 A◦), Ser 203 (2.94 A◦), Ser 293 (2.38 A◦), His 447 (2.05 A◦); *Carbon-hydrogen*: Trp 86 (3.49 A◦); *Electrostatic attraction*: Asp 74 (2.91 A◦, 2.84 A◦); *Pi- sulfur*: His 447 (4.57 A◦); *Hydrophobic interactions: Van der waals; Pi- alkyl*: Phe 338 (5.18 A◦), Tyr 341 (4.40 A◦); *Pi- pi*: Trp 286 (5.04 A◦,5.52 A◦ *); Pi- sigma* : Leu 289 (3.90 A◦).
**4r**	− 11.7	*Hydrogen bond*: Gly 121 (2.41 A◦), Gly 122 (2.40 A◦, 2.63 A◦), Ser 203 (2.88 A◦), Ser 293 (2.41 A◦); *Carbon-hydrogen*: Glu 202 (3.58 A◦); *Electrostatic attraction*: Asp 74 (2.91 A◦, 3.17 A◦); *Pi- sulfur*: Phe 338 (5.24 A◦); *Hydrophobic interactions: Van der waals; Pi- alkyl*: Trp 86 (4.93 A◦), His 447 (4.65 A◦), Leu 289 (5.14 A◦), Phe 297(4.93 A◦), Phe 338 (4.93 A◦), Tyr 341 (4.43 A◦); *Pi- pi*: Trp 286 (5.83 A◦), Tyr 341 (5.58 A◦)
**Donepezil**	− 10.8	NA

A closer look at Fig. 6 and detailed binding interactions in Table 6 revealed that in all of our compounds, benzimidazole NH, were predicted to form a hydrogen bond with Ser 293. Additionally, the sulfonate group SO_2_ played a key role in binding affinity in CAS because it formed four hydrogen bonds with Gly 121, Gly 122, Ser 203, and His 447 residues in compounds **4b** and **4q**. In compound **4r**, it formed three hydrogen bonds with Gly 121, Gly 122, and Ser 203, while compounds **4h** and **4i** formed two hydrogen bonds with Ser 203 and His 447 or Gly 121 and His 447, respectively. Furthermore, the sulfur atom engaged in Pi-sulfur interactions with His 447 in compounds **4i**, **4h**, and **4r**, and with Phe 338 in **4b** and **4q**. Adding van der Waals interactions to the previously mentioned bonds further strengthened the binding of the tested compounds in the CAS of AChE. In compounds **4b**, **4h**, **4i**, **4r**, and **4q**, it was observed that the aromatic ring of the benzimidazole group formed a hydrophobic Pi-sigma interaction with Leu 289 residue, representing a new region in PAS not reported in donepezil. Conversely, the aryl rings A formed a hydrophobic Pi-pi interaction with Trp 286, similar to the interaction with the fused aryl ring in donepezil. Additionally, the hydrazono group in the carbohydrazide linker formed an electrostatic attraction with Asp 74. Focusing on the linker, in compound **4i**, it established two hydrogen bonds with Try 124 and Phe 295, whereas in compound **4r**, it formed a single hydrogen bond with Try 124. The high binding affinity of compounds **4q** and **4r** might be due to the methoxy group attached to the aryl ring A, which created Pi-alkyl interactions with Tyr 341 and Phe 338.

To sum up, the docking study was in agreement with the in vitro AChE inhibitory assessment and clarified the slight differences in the biochemical results.

## Experimental

### Chemistry

General procedure for the synthesis of compounds **4a-r**.

Equimolar amounts of 2-(4-(1*H*-benzo[d]imidazol-2-yl)phenoxy)acetohydrazide derivative **2a**,** b** (0.01 mol) and suitable alkane phenyl sulfonate **3a-i** (0.01 mol) in ethanol (20 mL) with a few drops of glacial acetic acid were refluxed for 3 h. The solvent was then removed, and the reaction mixture was poured into ice-cold water. The crude product obtained was filtered, dried, and crystallized from ethanol.

*4-((2-(2-(4-(1 H-benzo[d]imidazol-2-yl)phenoxy)acetyl)hydrazono)methyl)phenyl methanesulfonate* (**4a**).

Yield: 89%; mp 208–210 °C; IR (KBr) cm^−1^, ν: 3325 (OH, tautomer), 3170 (NH), 1671 (C=O), 1620 (C=N), 1355 (SO_2_), 1214 (C–O, C–N); ^1^H NMR δ (ppm): 3.42 (s, 3 H, CH_3_), 4.89 (s, 2 H, CH_2_, iminol tautomer), 5.34 (s, 2 H, CH_2_, amide tautomer), 7.27 (d, 1H, *J* = 9.0 Hz, H_ar), 7.31 (d, 1H, *J* = 9.5 Hz, H_ar), 7.43 (d, 2 H, *J* = 8.5 Hz, H_ar), 7.49 (d, 2 H, *J* = 8.5 Hz, H_ar), 7.78 (d, 2 H, *J* = 9.0 Hz, H_ar), 7.86 (d, 2 H, *J* = 8.5 Hz, H_ar), 8.09 (s, 1H, CH=N, iminol tautomer), 8.35 (d, 1H, *J* = 8.0 Hz, H_ar), 8.37 (d, 1H, *J* = 8.5 Hz, H_ar), 8.74 (s, 1H, CH=N, amide tautomer), 11.81 (s, 1H, NH), 12.03 (s, 1H, NH); ^13^C NMR δ (ppm): 37.61, 65.12 (iminol tautomer), 66.38 (amide tautomer), 113.77, 115.75, 122.69, 125.33, 128.66, 128.81, 129.93, 130.16, 132.16, 132.32, 133.11, 142.68, 146.63, 148.76, 149.95, 151.02, 160.44, 161.52, 162.09, 163.85 (C_ar), 168.51 (CO); Anal. calc. for C_23_H_20_N_4_O_5_S (464.50): % C, 59.47; H, 4.34; N, 12.06; Found: % C, 59.55; H, 4.22; N, 12.15.

*4-((2-(2-(4-(1 H-benzo[d]imidazol-2-yl)phenoxy)acetyl)hydrazono)methyl)phenyl ethanesulfonate* (**4b**).

Yield: 81%; mp 266–268 °C; IR (KBr) cm^−1^, ν: 3317 (OH, tautomer), 3168 (NH), 1679 (C=O), 1630 (C=N), 1360 (SO_2_), 1210 (C–O, C–N); ^1^H NMR δ (ppm): 1.38 (t, 3 H, *J* = 7.0 Hz, CH_3_), 3.57 (q, 2 H, *J* = 6.5 Hz, CH_2_), 4.86 (s, 2 H, CH_2_, iminol tautomer), 5.33 (s, 2 H, CH_2_, amide tautomer), 7.24 (d, 1H, *J* = 10.5 Hz, H_ar), 7.27 (d, 1H, *J* = 9.0 Hz, H_ar), 7.40–7.41 (m, 2 H, H_ar), 7.47 (d, 1H, *J* = 8.5 Hz, H_ar), 7.71–7.73 (m, 2 H, H_ar), 7.80 (d, 1H, *J* = 9.0 Hz, H_ar), 7.85 (d, 1H, *J* = 6.0 Hz, H_ar), 7.99 (d, 1H, *J* = 6.0 Hz, H_ar), 8.08 (s, 1H, CH=N, iminol tautomer), 8.27–8.30 (m, 2 H, H_ar), 8.41 (s, 1H, H_ar), 8.75 (s, 1H, CH=N, amide tautomer), 11.79 (s, 1H, NH), 11.96 (s, 1H, NH); ^13^C NMR δ (ppm): 8.07 (CH_3_), 44.84 (CH_2_), 65.06 (iminol tautomer), 66.41 (amide tautomer), 99.44, 114.10, 115.57, 117.60, 118.49, 122.58, 124.45, 128.66, 128.82, 129.43, 130.17, 133.03, 134.02, 134.42, 142.69, 146.63, 149.46, 149.81, 160.71, 161.49 (C_ar), 168.63 (CO); Anal. calc. for C_24_H_22_N_4_O_5_S (478.52): % C, 60.24; H, 4.63; N, 11.71; Found: % C, 60.32; H, 4.51; N, 11.60.

*4-((2-(2-(4-(1 H-benzo[d]imidazol-2-yl)phenoxy)acetyl)hydrazono)methyl)phenyl propane-1-sulfonate* (**4c**).

Yield: 79%; mp 274–276 °C; IR (KBr) cm^−1^, ν: 3310 (OH, tautomer), 3185 (NH), 1672 (C=O), 1636 (C=N), 1345 (SO_2_), 1218 (C–O, C–N); ^1^H NMR δ (ppm): 1.03 (t, 3 H, *J* = 6.0 Hz, CH_3_), 1.85 (sextet, 2 H, *J* = 5.5 Hz, CH_2_*CH*_*2*_CH_3_), 3.55 (t, 2 H, *J* = 6.0 Hz, *CH*_*2*_CH_2_CH_3_), 4.89 (s, 2 H, CH_2_, iminol tautomer), 5.34 (s, 2 H, CH_2_, amide tautomer), 7.27 (d, 1H, *J* = 9.0 Hz, H_ar), 7.32 (d, 1H, *J* = 8.5 Hz, H_ar), 7.40 (d, 1H, *J* = 8.5 Hz, H_ar), 7.46 (d, 1H, *J* = 8.5 Hz, H_ar), 7.50 (dd, 2 H, *J* = 5.5, 3.0 Hz, H_ar), 7.79 (dd, 2 H, *J* = 5.5, 3.0 Hz, H_ar), 7.85 (d, 1H, *J* = 9.0 Hz, H_ar), 7.98 (d, 1H, *J* = 8.5 Hz, H_ar), 8.09 (s, 1H, CH=N, iminol tautomer), 8.37 (d, 1H, *J* = 9.0 Hz, H_ar), 8.41 (d, 1H, *J* = 9.5 Hz, H_ar), 8.73 (s, 1H, CH=N, amide tautomer), 11.81 (s, 1H, NH), 12.05 (s, 1H, NH); ^13^C NMR δ (ppm): 12.37 (CH_3_), 17.01 (CH_2_*CH*_*2*_CH_3_), 51.55 (*CH*_*2*_CH_2_CH_3_), 65.11 (iminol tautomer), 66.39 (amide tautomer), 99.46, 113.78, 115.67, 116.05, 122.59, 125.18, 128.66, 128.81, 129.85, 130.17, 133.00, 142.71, 146.66, 148.89, 149.79, 150.86, 160.44, 161.40, 162.00, 163.87 (C_ar), 168.53 (CO); Anal. calc. for C_25_H_24_N_4_O_5_S (492.55): % C, 60.96; H, 4.91; N, 11.38; Found: % C, 60.85; H, 4.80; N, 11.26.

*4-((2-(2-(4-(1 H-benzo[d]imidazol-2-yl)phenoxy)acetyl)hydrazono)methyl)-2-methoxy phenyl methanesulfonate* (**4d**).

Yield: 80%; mp 256–258 °C; IR (KBr) cm^−1^, ν: 3321 (OH, tautomer), 3173 (NH), 1673 (C=O), 1622 (C=N), 1360 (SO_2_), 1210 (C–O, C–N); ^1^H NMR δ (ppm): 3.37 (s, 3 H, CH_3_), 3.91 (s, 3 H, OCH_3_), 4.85 (s, 2 H, CH_2_, iminol tautomer), 5.33 (s, 2 H, CH_2_, amide tautomer), 7.21 (d, 1H, *J* = 14.0 Hz, H_ar), 7.26 (d, 1H, *J* = 14.0 Hz, H_ar), 7.33–7.38 (m, 3 H, H_ar), 7.44 (d, 1H, *J* = 14.0 Hz, H_ar), 7.52 (d, 1H, *J* = 14.0 Hz, H_ar), 7.68–7.70 (m, 2 H, H_ar), 8.05 (s, 1H, CH=N, iminol tautomer), 8.22–8.27 (m, 2 H, H_ar), 8.73 (s, 1H, CH=N, amide tautomer), 11.79 (s, 1H, NH), 11.92 (s, 1H, NH); ^13^C NMR δ (ppm): 38.48 (CH_3_), 56.11 (OCH_3_), 66.08 (iminol tautomer), 66.41 (amide tautomer), 111.26, 112.00, 114.17, 115.47, 119.80, 121.84, 123.82, 124.00, 124.24, 129.10, 134.21, 135.01, 138.01, 138.88, 142.78, 149.88, 151.74, 160.75, 161.17 (C_ar), 168.79 (CO); Anal. calc. for C_24_H_22_N_4_O_6_S (494.52): % C, 58.29; H, 4.48; N, 11.33; Found: % C, 58.38; H, 4.57; N, 11.20.

*4-((2-(2-(4-(1 H-benzo[d]imidazol-2-yl)phenoxy)acetyl)hydrazono)methyl)-2-methoxy phenyl ethanesulfonate* (**4e**).

Yield: 83%; mp 268–270 °C; IR (KBr) cm^−1^, ν: 3327 (OH, tautomer), 3188 (NH), 1669 (C=O), 1623 (C=N), 1359 (SO_2_), 1225 (C–O, C–N); ^1^H NMR δ (ppm): 1.39 (t, 3 H, *J* = 12.0 Hz, CH_3_), 3.51 (q, 2 H, *J* = 6.5 Hz, CH_2_), 3.91 (s, 3 H, OCH_3_), 4.90 (s, 2 H, CH_2_, iminol tautomer), 5.37 (s, 2 H, CH_2_, amide tautomer), 7.27 (d, 2 H, *J* = 14.5 Hz, H_ar), 7.34–7.35 (m, 2 H, H_ar), 7.50 (dd, 2 H, *J* = 10.5, 5.0 Hz, H_ar), 7.53 (s, 1H, H_ar), 7.79 (dd, 2 H, *J* = 10.5, 5.0 Hz, H_ar), 8.06 (s, 1H, CH=N, iminol tautomer), 8.34 (d, 2 H, *J* = 14.0 Hz, H_ar), 8.40 (s, 1H, CH=N, amide tautomer), 11.82 (s, 1H, NH), 12.02 (s, 1H, NH); ^13^C NMR δ (ppm): 8.17 (CH_3_), 45.83 (CH_2_), 56.19 (OCH_3_), 65.22 (iminol tautomer), 66.22 (amide tautomer), 110.81, 111.24, 113.87, 115.74, 115.93, 119.84, 120.45, 124.27, 125.37, 129.93, 132.38, 132.55, 134.09, 138.86, 139.07, 142.94, 148.93, 151.73, 161.50, 162.14, 164.00 (C_ar), 168.71 (CO); Anal. calc. for C_25_H_24_N_4_O_6_S (508.55): % C, 59.05; H, 4.76; N, 11.02; Found: % C, 59.16; H, 4.67; N, 11.13.

*4-((2-(2-(4-(1 H-benzo[d]imidazol-2-yl)phenoxy)acetyl)hydrazono)methyl)-2-methoxy phenylpropane-1-sulfonate* (**4f**).

Yield: 75%; mp 149–151 °C; IR (KBr) cm^−1^, ν: 3314 (OH, tautomer), 3187 (NH), 1683 (C=O), 1624 (C=N), 1366 (SO_2_), 1220 (C–O, C–N); ^1^H NMR δ (ppm): 1.04 (t, 3 H, *J* = 12.5 Hz, CH_3_), 1.87 (sextet, 2 H, *J* = 12.5 Hz, CH_2_*CH*_*2*_CH_3_), 3.48 (t, 2 H, *J* = 12.5 Hz, *CH*_*2*_CH_2_CH_3_), 3.90 (s, 3H, OCH_3_), 4.79 (s, 2 H, CH_2_, iminol tautomer), 5.28 (s, 2 H, CH_2_, amide tautomer), 7.12 (d, 2 H, *J* = 14.5 Hz, H_ar), 7.17 (dd, 2 H, *J* = 10.0, 5.0 Hz, H_ar), 7.33–7.38 (m, 2 H, H_ar), 7.49 (s, 1H, H_ar), 7.56 (dd, 2 H, *J* = 10.0, 5.0 Hz, H_ar), 8.03 (s, 1H, CH=N, iminol tautomer), 8.09–8.16 (m, 2 H, H_ar), 8.36 (s, 1H, CH=N, amide tautomer), 11.69 (s, 1H, NH), 11.74 (s, 1H, NH); ^13^C NMR δ (ppm): 12.49 (CH_3_), 17.15 (CH_2_*CH*_*2*_CH_3_), 52.50 (*CH*_*2*_CH_2_CH_3_), 56.14 (OCH_3_), 65.00 (iminol tautomer), 66.55 (amide tautomer), 110.71, 111.14, 115.05, 115.24, 119.88, 120.56, 121.99, 122.79, 123.39, 124.34, 128.06, 134.10, 136.86, 139.11, 142.79, 147.09, 151.35, 151.73, 159.20, 159.77, 164.34 (C_ar), 169.12 (CO); Anal. calc. for C_26_H_26_N_4_O_6_S (522.58): % C, 59.76; H, 5.02; N, 10.72; Found: % C, 59.84; H, 5.16; N, 10.86.

*5-((2-(2-(4-(1 H-benzo[d]imidazol-2-yl)phenoxy)acetyl)hydrazono)methyl)-2-methoxy phenyl methanesulfonate* (**4g**).

Yield: 85%; mp 215–217 °C; IR (KBr) cm^−1^, ν: 3316 (OH, tautomer), 3185 (NH), 1670 (C=O), 1630 (C=N), 1349 (SO_2_), 1207 (C–O, C–N); ^1^H NMR δ (ppm): 3.39 (s, 3 H, CH_3_), 3.87 (s, 3 H, OCH_3_), 4.87 (s, 2 H, CH_2_, iminol tautomer), 5.29 (s, 2 H, CH_2_, amide tautomer), 7.21 (d, 1H, *J* = 13.0 Hz, H_ar), 7.24–7.28 (m, 2 H, H_ar), 7.42–7.45 (m, 2 H, H_ar), 7.62–7.67 (m, 2 H, H_ar), 7.73–7.74 (m, 2 H, H_ar), 8.03 (s, 1H, CH=N, iminol tautomer), 8.32 (s, 1H, CH=N, amide tautomer), 8.36 (d, 2 H, *J* = 10.5 Hz, H_ar), 11.74 (s, 1H, NH), 12.05 (s, 1H, NH); ^13^C NMR δ (ppm): 38.45 (CH_3_), 56.32 (OCH_3_), 65.17 (iminol tautomer), 66.40 (amide tautomer), 113.84, 115.70, 116.03, 116.75, 121.74, 125.22, 127.15, 127.58, 127.96, 127.88, 132.51, 132.73, 138.19, 142.85, 146.76, 148.87, 152.83, 161.41, 162.06, 163.81 (C_ar), 168.45 (CO); Anal. calc. for C_24_H_22_N_4_O_6_S (494.52): % C, 58.29; H, 4.48; N, 11.33; Found: % C, 58.17; H, 4.33; N, 11.21.

*5-((2-(2-(4-(1 H-benzo[d]imidazol-2-yl)phenoxy)acetyl)hydrazono)methyl)-2-methoxy phenyl ethanesulfonate* (**4h**).

Yield: 82%; mp 152–154 °C; IR (KBr) cm^−1^, ν: 3319 (OH, tautomer), 3189 (NH), 1682 (C=O), 1622 (C=N), 1360 (SO_2_), 1219 (C–O, C–N);^1^H NMR δ (ppm): 1.40 (t, 3 H, *J* = 12.5 Hz, CH_3_), 3.53 (q, 2 H, *J* = 12.0 Hz, CH_2_), 3.89 (s, 3 H, OCH_3_), 4.81 (s, 2 H, CH_2_, iminol tautomer), 5.29 (s, 2 H, CH_2_, amide tautomer), 7.18 (d, 1H, *J* = 14.5 Hz, H_ar), 7.23–7.28 (m, 2 H, H_ar), 7.33 (dd, 2 H, *J* = 10.0, 5.0 Hz, H_ar), 7.64–7.68 (m, 4 H, H_ar), 8.00 (s, 1H, CH=N, iminol tautomer), 8.18–8.23 (m, 2 H, H_ar), 8.32 (s, 1H, CH=N, amide tautomer), 11.65 (s, 1H, NH), 11.75 (s, 1H, NH); ^13^C NMR δ (ppm): 8.17 (CH_3_), 45.86 (CH_2_), 56.34 (OCH_3_), 65.04 (iminol tautomer), 66.45 (amide tautomer), 113.72, 114.36, 114.47, 115.40, 115.49, 121.69, 123.46, 123.67, 127.14, 127.50, 127.88, 128.92, 135.91, 136.42, 138.11, 142.79, 150.25, 152.84, 160.95, 163.97 (C_ar), 168.63 (CO); Anal. calc. for C_25_H_24_N_4_O_6_S (508.55): % C, 59.05; H, 4.76; N, 11.02; Found: % C, 59.14; H, 4.67; N, 11.11.

*5-((2-(2-(4-(1 H-benzo[d]imidazol-2-yl)phenoxy)acetyl)hydrazono)methyl)-2-methoxy phenyl propane-1-sulfonate* (**4i**).

Yield: 78%; mp 226–228 °C; IR (KBr) cm^−1^, ν: 3311 (OH, tautomer), 3185 (NH), 1683 (C=O), 1637 (C=N), 1358 (SO_2_), 1215 (C–O, C–N); ^1^H NMR δ (ppm): 1.03 (t, 3 H, *J* = 7.5 Hz, CH_3_), 1.87 (sextet, 2 H, *J* = 7.5 Hz, CH_2_*CH*_*2*_CH_3_), 3.50 (t, 2 H, *J* = 7.5 Hz, *CH*_*2*_CH_2_CH_3_), 3.89 (s, 3 H, OCH_3_), 4.87 (s, 2 H, CH_2_, iminol tautomer), 5.33 (s, 2 H, CH_2_, amide tautomer), 7.26 (d, 1H, *J* = 9.0 Hz, H_ar), 7.28–7.32 (m, 2 H, H_ar), 7.48 (dd, 2 H, *J* = 9.5, 5.0 Hz, H_ar), 7.63 (s, 1H, H_ar), 7.66–7.67 (m, 2 H, H_ar), 7.77 (dd, 2 H, *J* = 9.5, 5.0 Hz, H_ar), 8.01 (s, 1H, CH=N, iminol tautomer), 8.35 (d, 1H, *J* = 9.0 Hz, H_ar), 8.38 (d, 1H, *J* = 8.5 Hz, H_ar), 8.68 (s, 1H, CH=N, amide tautomer), 11.72 (s, 1H, NH), 11.95 (s, 1H, NH); ^13^C NMR δ (ppm): 12.41 (CH_3_), 17.07 (CH_2_*CH*_*2*_CH_3_), 52.47 (*CH*_*2*_CH_2_CH_3_), 56.25 (OCH_3_), 65.10 (iminol tautomer), 66.36 (amide tautomer), 99.44, 113.77, 115.61, 121.68, 125.25, 127.07, 129.97, 132.24, 137.92, 138.02, 142.74, 146.65, 148.72, 152.72, 152.85, 153.75, 160.06, 161.48, 162.09, 163.65 (C_ar), 168.30 (CO); Anal. calc. for C_26_H_26_N_4_O_6_S (522.58): % C, 59.76; H, 5.02; N, 10.72; Found: % C, 59.61; H, 5.14; N, 10.86.

*4-((2-(2-(4-(1 H-benzo[d]imidazol-2-yl)-2-methoxyphenoxy)acetyl)hydrazono)methyl) phenyl methanesulfonate* (**4j**).

Yield: 90%; mp 172–174 °C; IR (KBr) cm^−1^, ν: 3324 (OH, tautomer), 3171 (NH), 1670 (C=O), 1620 (C=N), 1356 (SO_2_), 1213 (C–O, C–N); ^1^H NMR δ (ppm): 3.42 (s, 3 H, CH_3_), 3.92 (s, 3 H, OCH_3_), 4.77 (s, 2 H, CH_2_, iminol tautomer), 5.27 (s, 2 H, CH_2_, amide tautomer), 7.09 (d, 1H, *J* = 9.0 Hz, H_ar), 7.22–7.23 (m, 2 H, H_ar), 7.41–7.43 (m, 2 H, H_ar), 7.59–7.60 (m, 2 H, H_ar), 7.70–7.74 (m, 2 H, H_ar), 7.81–7.85 (m, 2 H, H_ar), 8.00 (d, 1H, *J* = 9.0 Hz, H_ar), 8.05 (s, 1H, CH=N, iminol tautomer), 8.34 (s, 1H, CH=N, amide tautomer), 11.73 (s, 1H, NH); ^13^C NMR δ (ppm): 37.56 (CH_3_), 56.77 (OCH_3_), 65.23 (iminol tautomer), 67.39 (amide tautomer), 110.38, 113.19, 114.61, 119.46, 122.36, 122.70, 128.64, 128.83, 130.19, 133.22, 138.37, 142.51, 146.50, 149.04, 149.59, 149.96, 151.08, 160.49, 164.24, 167.29 (C_ar), 168.85 (CO); Anal. calc. for C_24_H_22_N_4_O_6_S (494.52): % C, 58.29; H, 4.48; N, 11.33; Found: % C, 58.42; H, 4.31; N, 11.21.

*4-((2-(2-(4-(1 H-benzo[d]imidazol-2-yl)-2-methoxyphenoxy)acetyl)hydrazono)methyl) phenyl ethanesulfonate* (**4k**).

Yield: 88%; mp 110–112 °C; IR (KBr) cm^−1^, ν: 3320 (OH, tautomer), 3175 (NH), 1672 (C=O), 1622 (C=N), 1361 (SO_2_), 1212 (C–O, C–N); ^1^H NMR δ (ppm): 1.38 (t, 3 H, *J* = 14.0 Hz, CH_3_), 3.55 (q, 2 H, *J* = 14.5 Hz, CH_2_), 3.93 (s, 3 H, OCH_3_), 4.78 (s, 2 H, CH_2_, iminol tautomer), 5.28 (s, 2 H, CH_2_, amide tautomer), 7.08 (d, 1H, *J* = 14.0 Hz, H_ar), 7.23–7.25 (m, 2 H, H_ar), 7.40 (d, 1H, *J* = 14.5 Hz, H_ar), 7.46 (d, 1H, *J* = 14.5 Hz, H_ar), 7.60–7.63 (m, 2 H, H_ar), 7.73–7.77 (m, 1H, H_ar), 7.81–7.86 (m, 2 H, H_ar), 7.78 (d, 1H, *J* = 14.5 Hz, H_ar), 8.06 (s, 1H, CH=N, iminol tautomer), 8.35 (s, 1H, CH=N, amide tautomer), 11.70 (s, 1H, NH); ^13^C NMR δ (ppm): 8.16 (CH_3_), 44.93 (CH_2_), 56.87 (OCH_3_), 65.32 (iminol tautomer), 67.36 (amide tautomer), 110.51, 113.26, 114.05, 114.64, 119.76, 121.48, 122.68, 128.73, 128.94, 130.27, 133.11, 137.81, 142.69, 146.64, 149.14, 149.37, 149.87, 151.00, 160.59, 164.38 (C_ar), 168.93 (CO); Anal. calc. for C_25_H_24_N_4_O_6_S (508.55): % C, 59.04; H, 4.76; N, 11.02; Found: % C, 59.11; H, 4.67; N, 11.16.

*4-((2-(2-(4-(1 H-benzo[d]imidazol-2-yl)-2-methoxyphenoxy)acetyl)hydrazono)methyl) phenyl propane-1-sulfonate* (**4l **).

Yield: 78%; mp 145–147 °C; IR (KBr) cm^−1^, ν: 3318 (OH, tautomer), 3167 (NH), 1678 (C=O), 1630 (C=N), 1362 (SO_2_), 1211 (C–O, C–N); ^1^H NMR δ (ppm): 1.04 (t, 3 H, *J* = 7.5 Hz, CH_3_), 1.87 (sextet, 2 H, *J* = 7.5 Hz, CH_2_*CH*_*2*_CH_3_), 3.51 (t, 2 H, *J* = 7.5 Hz, *CH*_*2*_CH_2_CH_3_), 3.94 (s, 3 H, OCH_3_), 4.27 (s, 2 H, CH_2_, iminol tautomer), 5.28 (s, 2 H, CH_2_, amide tautomer), 7.07 (d, 1H, *J* = 10.0 Hz, H_ar), 7.32–7.24 (m, 2 H, H_ar), 7.42–7.44 (m, 2 H, H_ar), 7.58–7.60 (m, 2 H, H_ar), 7.71–7.75 (m, 2 H, H_ar), 7.80–7.84 (m, 2 H, H_ar), 8.01 (d, 1H, *J* = 10.0 Hz, H_ar), 8.05 (s, 1H, CH=N, iminol tautomer), 8.33 (s, 1H, CH=N, amide tautomer), 11.74 (s, 1H, NH); ^13^C NMR δ (ppm): 12.41 (CH_3_), 17.05 (CH_2_*CH*_*2*_CH_3_), 51.53 (*CH*_*2*_CH_2_CH_3_), 55.81 (OCH_3_), 65.25 (iminol tautomer), 67.33 (amide tautomer), 110.45, 113.20, 114.00, 114.57, 119.69, 121.39, 122.64, 128.67, 128.86, 130.21, 133.04, 137.73, 138.09, 142.59, 146.55, 149.07, 149.31, 149.80, 149.96, 150.91, 156.42, 164.28 (C_ar), 168.84 (CO); Anal. calc. for C_26_H_26_N_4_O_6_S (522.57): % C, 59.76; H, 5.01; N, 10.72; Found: % C, 59.60; H, 5.17; N, 10.83.

*4-((2-(2-(4-(1 H-benzo[d]imidazol-2-yl)-2-methoxyphenoxy)acetyl)hydrazono)methyl)-2-methoxyphenyl methanesulfonate* (**4m**).

Yield: 91%; mp 160–162 °C; IR (KBr) cm^−1^, ν: 3325 (OH, tautomer), 3187 (NH), 1670 (C=O), 1623 (C=N), 1358 (SO_2_), 1223 (C–O, C–N); ^1^H NMR δ (ppm): 3.40 (s, 3 H, SO_2_CH_3_), 3.91 (s, 3 H, OCH_3_), 3.94 (s, 3 H, OCH_3_), 4.76 (s, 2 H, CH_2_, iminol tautomer), 5.28 (s, 2 H, CH_2_, amide tautomer), 7.04 (d, 1H, *J* = 8.5 Hz, H_ar), 7.18–7.20 (m, 2 H, H_ar), 7.33–7.38 (m, 2 H, H_ar), 7.53 (s, 1H, H_ar), 7.57–7.59 (m, 2 H, H_ar), 7.69–7.73 (m, 2 H, H_ar), 7.80–7.83 (m, 1H, H_ar), 8.02 (s, 1H, CH=N, iminol tautomer), 8.31 (s, 1H, CH=N, amide tautomer), 11.75 (s, 1H, NH); ^13^C NMR δ (ppm): 21.02 (CH_3_), 38.44 (CH_3_), 55.73, 56.09 (OCH_3_), 65.27 (iminol tautomer), 67.43 (amide tautomer), 110.26, 111.20, 111.12, 114.09, 119.24, 119.76, 122.04, 122.63, 124.22, 134.15, 138.86, 140.02, 142.61, 148.99, 149.38, 151.72, 160.74, 164.27 (C_ar), 168.98, 171.99 (CO); Anal. calc. for C_25_H_24_N_4_O_7_S (524.55): % C, 57.24; H, 4.61; N, 10.68; Found: % C, 57.12; H, 4.50; N, 10.55.

*4-((2-(2-(4-(1 H-benzo[d]imidazol-2-yl)-2-methoxyphenoxy)acetyl)hydrazono)methyl)-2-methoxyphenyl ethanesulfonate* ***(4n).***

Yield: 86%; mp 140–142 °C; IR (KBr) cm^−1^, ν: 3317 (OH, tautomer), 3186 (NH), 1675 (C=O), 1630 (C=N), 1347 (SO_2_), 1206 (C–O, C–N); ^1^H NMR δ (ppm): 1.39 (t, 3 H, *J* = 12.5 Hz, CH_3_), 3.50 (q, 2 H, *J* = 12.0 Hz, CH_2_), 3.90 (s, 3 H, OCH_3_), 3.94 (s, 3 H, OCH_3_), 4.77 (s, 2 H, CH_2_, iminol tautomer), 5.28 (s, 2 H, CH_2_, amide tautomer), 7.06 (d, 1H, *J* = 14.0 Hz, H_ar), 7.20–7.23 (m, 2 H, H_ar), 7.32–7.38 (m, 2 H, H_ar), 7.49–7.52 (m, 1H, H_ar), 7.58–7.61 (m, 2 H, H_ar), 7.71 (d, 1H, *J* = 14.0 Hz, H_ar), 7.83 (d, 1H, *J* = 14.0 Hz, H_ar), 8.02 (s, 1H, CH=N, iminol tautomer), 8.31 (s, 1H, CH=N, amide tautomer), 11.73 (s, 1H, NH); ^13^C NMR δ (ppm): 8.13 (CH_3_), 21.08 (OCH_3_), 45.86 (CH_2_), 55.38, 56.10 (OCH_3_), 65.31 (iminol tautomer), 67.39 (amide tautomer), 110.36, 111.15, 111.95, 113.16, 114.71, 119.46, 119.80, 120.46, 122.36, 124.25, 134.10, 138.83, 142.71, 146.84, 149.08, 149.62, 151.17, 151.74, 164.34 (C_ar), 169.01, 172.12 (CO); Anal. calc. for C_26_H_26_N_4_O_7_S (538.57): % C, 57.98; H, 4.87; N, 10.40; Found: % C, 57.81; H, 4.71; N, 10.56.

*4-((2-(2-(4-(1 H-benzo[d]imidazol-2-yl)-2-methoxyphenoxy)acetyl)hydrazono)methyl)-2-methoxyphenyl propane-1-sulfonate* (**4o**).

Yield: 80%; mp 120–122 °C; IR (KBr) cm^−1^, ν: 3316 (OH, tautomer), 3180 (NH), 1680 (C=O), 1622 (C=N), 1361 (SO_2_), 1217 (C–O, C–N); ^1^H NMR δ (ppm): 1.03 (t, 3 H, *J* = 7.5 Hz, CH_3_), 1.87 (sextet, 2 H, *J* = 7.5 Hz, CH_2_*CH*_*2*_CH_3_), 3.48 (t, 2 H, *J* = 8.0 Hz, *CH*_*2*_CH_2_CH_3_), 3.90 (s, 3 H, OCH_3_), 3.93 (s, 3 H, OCH_3_), 4.77 (s, 2 H, CH_2_, iminol tautomer), 5.29 (s, 2 H, CH_2_, amide tautomer), 7.05 (d, 1H, *J* = 8.5 Hz, H_ar), 7.21–7.22 (m, 2 H, H_ar), 7.32–7.35 (m, 2 H, H_ar), 7.49–7.53 (m, 1H, H_ar), 7.58–7.60 (m, 2 H, H_ar), 7.70–7.74 (m, 1H, H_ar), 7.82 (d, 1H, *J* = 12.0 Hz, H_ar), 8.02 (s, 1H, CH=N, iminol tautomer), 8.30 (s, 1H, CH=N, amide tautomer), 11.76 (s, 1H, NH); ^13^C NMR δ (ppm): 12.45 (CH_3_), 17.11 (CH_2_*CH*_*2*_CH_3_), 21.05 (OCH_3_), 52.50 (*CH*_*2*_CH_2_CH_3_), 55.78, 56.10 (OCH_3_), 65.34 (iminol tautomer), 67.43 (amide tautomer), 110.40, 110.80, 111.15, 113.19, 114.67, 119.48, 119.81, 120.45, 122.35, 124.27, 134.08, 138.84, 139.07, 142.71, 146.87, 149.07, 149.62, 151.71, 164.35 (C_ar), 169.00, 172.07 (CO); Anal. calc. for C_27_H_28_N_4_O_7_S (552.60): % C, 58.68; H, 5.11; N, 10.14; Found: % C, 58.81; H, 54.21; N, 10.05.

*5-((2-(2-(4-(1 H-benzo[d]imidazol-2-yl)-2-methoxyphenoxy)acetyl)hydrazono)methyl)-2-methoxyphenyl methanesulfonate* (**4p**).

Yield: 89%; mp 140–142 °C; IR (KBr) cm^−1^, ν: 3312 (OH, tautomer), 3182 (NH), 1676 (C=O), 1636 (C=N), 1346 (SO_2_), 1219 (C–O, C–N);^1^H NMR δ (ppm): 3.40 (s, 3 H, CH_3_), 3.89 (s, 3 H, OCH_3_), 3.92 (s, 3 H, OCH_3_), 4.76 (s, 2 H, CH_2_, iminol tautomer), 5.26 (s, 2 H, CH_2_, amide tautomer), 7.05 (d, 1H, *J* = 8.5 Hz, H_ar), 7.20–7.21 (m, 2 H, H_ar), 7.24–7.26 (m, 2 H, H_ar), 7.58–7.60 (m, 2 H, H_ar), 7.65–7.66 (m, 2 H, H_ar), 7.70–7.75 (m, 2 H, H_ar), 7.82 (s, 1H, H_ar), 7.98 (s, 1H, CH=N, iminol tautomer), 8.26 (s, 1H, CH=N, amide tautomer), 11.64 (s, 1H, NH); ^13^C NMR δ (ppm): 21.06 (CH_3_), 38.44 (CH_3_), 55.81, 56.30 (OCH_3_), 65.28 (iminol tautomer), 67.43 (amide tautomer), 110.40,113.22, 113.77, 114.74, 119.42, 121.71, 122.27, 122.74, 123.38, 126.89, 127.17, 127.48, 127.79, 129.33, 138.19, 142.62, 146.57, 149.07, 149.57, 151.23, 152.82, 153.88, 160.14, 164.13 (C_ar), 168.75, 172.07 (CO); Anal. calc. for C_25_H_24_N_4_O_7_S (524.55): % C, 57.24; H, 4.61; N, 10.68; Found: % C, 57.36; H, 4.73; N, 10.80.

*5-((2-(2-(4-(1 H-benzo[d]imidazol-2-yl)-2-methoxyphenoxy)acetyl)hydrazono)methyl)-2-methoxyphenyl ethanesulfonate* (**4q**).

Yield: 85%; mp 200–202 °C; IR (KBr) cm^−1^, ν: 3313 (OH, tautomer), 3179 (NH), 1681 (C=O), 1624 (C=N), 1365 (SO_2_), 1218 (C–O, C–N); ^1^H NMR δ (ppm): 1.39 (t, 3 H, *J* = 7.0 Hz, CH_3_), 3.53 (q, 2 H, *J* = 7.5 Hz, CH_2_), 3.89 (s, 3 H, OCH_3_), 3.92 (s, 3 H, OCH_3_), 4.75 (s, 2 H, CH_2_, iminol tautomer), 5.26 (s, 2 H, CH_2_, amide tautomer), 7.05 (d, 1H, *J* = 8.5 Hz, H_ar), 7.22–7.23 (m, 1H, H_ar), 7.25–7.28 (m, 1H, H_ar), 7.33 (d, 1H, *J* = 8.5 Hz, H_ar), 7.60 (d, 1H, *J* = 7.0 Hz, H_ar), 7.65 (d, 2 H, *J* = 9.0 Hz, H_ar), 7.70–7.74 (m, 2 H, H_ar), 7.79–7.84 (m, 2 H, H_ar), 7.98 (s, 1H, CH=N, iminol tautomer), 8.26 (s, 1H, CH=N, amide tautomer), 11.64 (s, 1H, NH); ^13^C NMR δ (ppm): 8.10 (CH_3_), 21.06 (OCH_3_), 45.81 (CH_2_), 55.83, 56.30 (OCH_3_), 65.29 (iminol tautomer), 67.40 (amide tautomer), 110.46, 113.68, 114.71, 119.55, 121.73, 122.47, 123.05, 127.15, 127.38, 129.26, 138.10, 142.66, 146.61, 149.10, 149.71, 151.12, 152.93, 153.84, 160.17, 164.14 (C_ar), 168.71, 172.08 (CO); Anal. calc. for C_26_H_26_N_4_O_7_S (538.57): % C, 57.98; H, 4.87; N, 10.40; Found: % C, 57.86; H, 4.98; N, 10.29.

*5-((2-(2-(4-(1 H-benzo[d]imidazol-2-yl)-2-methoxyphenoxy)acetyl)hydrazono)methyl)-2-methoxyphenyl propane-1-sulfonate* (**4r**).

Yield: 77%; mp 107–109 °C; IR (KBr) cm^−1^, ν: 3312 (OH, tautomer), 3188 (NH), 1684 (C=O), 1637 (C=N), 1356 (SO_2_), 1216 (C–O, C–N); ^1^H NMR δ (ppm): 1.03 (t, 3 H, *J* = 7.0 Hz, CH_3_), 1.86 (sextet, 2 H, *J* = 7.5 Hz, CH_2_*CH*_*2*_CH_3_), 3.50 (t, 2 H, *J* = 7.0 Hz, *CH*_*2*_CH_2_CH_3_), 3.89 (s, 3 H, OCH_3_), 3.92 (s, 3 H, OCH_3_), 4.75 (s, 2 H, CH_2_, iminol tautomer), 5.25 (s, 2 H, CH_2_, amide tautomer), 7.06 (d, 1H, *J* = 8.5 Hz, H_ar), 7.21–7.27 (m, 2 H, H_ar), 7.33 (d, 1H, *J* = 8.5 Hz, H_ar), 7.59–7.61 (m, 2 H, H_ar), 7.63–7.65 (m, 2 H, H_ar), 7.70–7.73 (m, 1H, H_ar), 7.79–7.84 (m, 2 H, H_ar), 7.98 (s, 1H, CH=N, iminol tautomer), 8.26 (s, 1H, CH=N, amide tautomer), 11.62 (s, 1H, NH); ^13^C NMR δ (ppm): 12.45 (CH_3_), 17.10 (CH_2_*CH*_*2*_CH_3_), 21.05 (OCH_3_), 52.30 (*CH*_*2*_CH_2_CH_3_), 55.83, 56.31 (OCH_3_), 65.27 (iminol tautomer), 67.43 (amide tautomer), 99.47, 110.49, 113.27, 113.68, 114.73, 119.62, 121.70, 122.57, 127.15, 127.40, 129.22, 138.08, 142.66, 146.59, 149.10, 149.36, 149.79, 151.05, 152.79, 152.92, 153.84, 160.16, 164.11 (C_ar), 168.69, 172.06 (CO); Anal. calc. for C_27_H_28_N_4_O_7_S (552.60): % C, 58.68; H, 5.11; N, 10.14; Found: % *C*,* 58.56; H*,* 5.27; N*,* 10.26.*

### Estimation of the inhibitory activity against acetylcholine esterase (AChE) and butyrylcholine esterase (BChE) enzymes

The assays were conducted using a 96-well plate, as reported previously^36–38^, and it is briefly described in the supporting information file.

### Antioxidant activity

1. *The 1*,*1-Diphenyl-2-picryl-hydrazyl (DPPH) radical scavenging assay*.
2. *The 2*,*2’-azinobis-(3-ethylbenzothiazoline-6-sulfonic acid) (ABTS) scavenging assay*.
3. *The nitrous oxide (NO) scavenging assay*.
4. *The hydroxyl (OH) radical scavenging assay*.
5. *The hydrogen peroxide (H*_*2*_*O*_*2*_*) radical scavenging assay*.

Antioxidant activity was determined using DPPH (1,1-diphenyl-2-picrylhydrazyl). The methodology was applied as described earlier^50–55^.

### Docking study

The grid box with a center at coordinates (x = 6.45, y = − 54.94, z = − 19.69 was defined to encompass the active site. The grid box dimensions were set to 22.13 Å × 25 Å × 21.8 Å to ensure full coverage of the binding pocket.

## Conclusion

In conclusion, this study successfully designed and synthesized a new series of benzimidazol-alkanesulfonate conjugates that demonstrate significant dual inhibitory activity against both acetylcholinesterase and butyrylcholinesterase, addressing a critical gap in Alzheimer’s disease treatment. Among the tested compounds, derivatives **4q** and **4r** exhibited the most promising results, showing potent AChE inhibition comparable to the established drug donepezil, as well as notable antioxidant properties. The in silico studies further support these findings, indicating strong binding affinities for the target enzymes. These results highlight the potential of these novel compounds as effective therapeutic agents for Alzheimer’s disease, warranting further investigation and development.

## Supplementary Information

Below is the link to the electronic supplementary material.

Supplementary Material 1

## Data Availability

All data generated or analyzed during this study are included in the manuscript and its supplementary information file.
