# Supplementary material for: New benzimidazole-alkanesulfonate conjugates as cholinesterase inhibitors with in vitro and in silico validation
Source: Sci Rep. 2026 Mar 12;16:8946. doi: 10.1038/s41598-026-39534-z (PMC12987925; doi:10.1038/s41598-026-39534-z)
Supplement: Supplementary file 1 — Supplementary Material 1 [file 41598_2026_39534_MOESM1_ESM.docx]

**Supplementary file**

**Design and Synthesis of new Benzimidazole-Alkanesulfonate Conjugates as Cholinesterase Inhibitors: *in vitro* and *in silico* Studies**

Mohamed A. Omar^a^, Aisha A.K. Al-Ashmawy^b^, Hayam A. Abd El Salam^c^, Riham A. El-Shiekh^d^, Wael Mahmoud Aboulthana^e^, Aladdin M. Srour^b,*^

^a^Chemistry of Natural and Microbial Products Department, Pharmaceutical and Drug Industries Research Institute, National Research Centre, Dokki, Giza, 12622, Egypt.

^b^Department of Therapeutic Chemistry, National Research Centre, Dokki, Giza, 12622, Egypt.

^c^Green Chemistry Department, Chemical Industries Research Institute, National Research Centre, Dokki, Giza, 12622, Egypt.

^d^Department of Pharmacognosy, Faculty of Pharmacy, Cairo University, Kasr el Aini St., 11562, Cairo, Egypt.

^e^Biochemistry Department, Biotechnology Research Institute, National Research Centre, 33 El Bohouth St., P.O. 12622, Dokki, Cairo, Egypt.

Corresponding author

Email address: [am.srour@nrc.sci.eg](mailto:am.srour@nrc.sci.eg)

1. ***In vitro biological activities***
   1. *Acetylcholine esterase (AChE) and butyrylcholine esterase (BChE) enzyme inhibition assays.*

The assays were performed using a 96-well plate, as previously reported. A Tecan microplate reader (USA) was employed. Bovine serum albumin was obtained from Sigma-Aldrich (St. Louis, MO, USA). Acetylcholinesterase (AChE) from electric eel (type VI-S lyophilized powder, EC 3.1.1.7) and horse butyrylcholinesterase (BChE) (EC 3.1.1.8) enzymes were purchased from Sigma-Aldrich (St. Louis, MO, USA). Butyrylthiocholine iodide and acetylthiocholine iodide were used as substrates in BChE and AChE assays, respectively, and were purchased from Sigma-Aldrich (St. Louis, MO, USA). Dithio-bis (2-nitrobenzoic acid) (DTNB) served as an indicator and was obtained from Sigma-Aldrich (St. Louis, MO, USA). Briefly, 170 *μ*L of 200 mM Tris-HCl buffer (pH 7.5) was added, followed by 20 *μ*L of test compounds at various concentrations (10-0.15625 *μ*g mL^−1^), and then 20 *μ*L of enzyme solution (0.1 U/mL). After incubating for 10 min at 25 °C, 40 *μ*L of DTNB was added, followed by 20 *μ*L of substrate (1.11 mM). All samples were dissolved in DMSO. The color intensity was measured at 405 nm with a microplate reader (reading A), and the control without inhibitor was measured (reading B). Blank assays replaced the enzyme with buffer, and their absorbances were recorded to correct for spontaneous indicator lysis or the inherent color of the inhibitor. All reactions were performed in triplicate. Linear regression was used to calculate the IC_50_ (50% inhibitory concentration) of active compounds only. Data analysis was conducted using Microsoft Excel 2010 (Redmond, WA, USA), and the percentage of inhibition was calculated with the following equation: % Inhibition = (1 - reading A/reading B) × 100.

- 1. *Antioxidant activity*

The total antioxidant capacity (TAC) was evaluated following the methods demonstrated by Prieto *et al*. (1999). It was determined by analyzing the green phosphate/Mo^5+^ complex at a wavelength (λ) of 695 nm. Samples were mixed with a reagent solution containing 0.3 N sulfuric acid, 28 mM sodium phosphate, and 4 mM ammonium molybdate. Methanol (80%) was used in place of the sample for the blank. The tubes were sealed and incubated in a boiling water bath for 90 minutes. After cooling to room temperature, the absorbance was measured at 695 nm against the blank. Ascorbic acid was used at the same concentrations as a standard. The antioxidant capacity was expressed as mg gallic acid equivalent per gram of weight.

The iron reducing power (IRP) was evaluated as µg/mL following the methods demonstrated by Oyaizu (1986). In brief, 1mL of the tested sample (at each concentration) was combined with 1mL of 200mM sodium phosphate buffer (pH 6.6) and 1mL of 1% potassium ferricyanide. The mixture was then incubated at 50°C for 20 minutes, followed by the addition of 1mL of trichloroacetic acid (10%). After centrifugation at 2000rpm for 10 minutes, the upper layer solution (2.5 mL) was mixed with 2.5 mL of double deionized water and 1mL of fresh ferric chloride (0.1%). The absorbance was measured at 700nm against a blank prepared without the sample. Ascorbic acid was used at the same concentrations as a standard. A high absorbance at 700nm indicates a higher reducing power in the reaction mixture.

***Scavenging activity***

- - 1. *The 1,1-Diphenyl-2-picryl-hydrazyl (DPPH) radical scavenging assay*

It was evaluated using the method proposed by Rahman *et al*. (2015). An antioxidant substance capable of donating a hydrogen atom to a solution containing DPPH- can reduce the stable free radical, causing the solution to change color from violet to pale yellow. The remaining DPPH- radical was quantified by measuring the intensity of a light-purple colored DPPH methanol solution in the visible range at 518 nm using a spectroscopic method. Two milliliters of a DPPH solution (100 *µ*M) in methanol were mixed with 2 mL of the sample (at each concentration). The reaction mixture for each concentration was thoroughly vortexed and then incubated in the dark at room temperature for 30 minutes. The absorbance was then measured spectrophotometrically at 518 nm against a blank (methanol). For the control, 2 mL of ethanol was added instead of the sample and run simultaneously with the test. Ascorbic acid was used at the same concentrations as a positive control. Percent inhibition of the DPPH free radical was calculated.

- - 1. *The 2,2'-azinobis-(3-ethylbenzothiazoline-6-sulfonic acid) (ABTS) scavenging assay*

During the procedure used for assaying the 2,2'-azinobis-(3-ethylbenzothiazoline-6-sulfonic acid) (ABTS) as demonstrated by Arnao *et al*. (2001), stock solutions included ABTS solution (7 mM) and potassium persulfate solution (2.4 mM). The working solution was prepared by mixing the two stock solutions in equal quantities and allowing them to react at room temperature in a dark place for 14 hours. The solution was then diluted by mixing 1 mL of ABTS solution with 60 mL of methanol to obtain an absorbance of 0.706 ± 0.01 units at 734 nm using a spectrophotometer. A fresh ABTS solution was prepared for each assay. The tested samples (at each concentration) were allowed to react with 1 mL of the ABTS solution, and the absorbance was taken at 734 nm after 7 minutes using a spectrophotometer. The ABTS scavenging capacities of the samples were compared with those of ascorbic acid (at the same concentrations).

- - 1. *The nitrous oxide (NO) scavenging assay*

In the assay concerned with evaluating the efficiency against nitrous oxide (NO) radicals as suggested by Chakraborthy (2009), Griess Illosvory reagent was generally modified by using napthyl ethylene diamine dihydrochloride (0.1% w/v) instead of 1-napthylamine (5%). The reaction mixture (3 mL) containing 2 mL of 10 mM sodium nitroprusside, 0.5 mL saline phosphate buffer and 0.5 mL of standard solution or tested samples (500–1000 μg/ mL) was incubated at 25°C for 150 minutes. After incubation, 0.5 mL of the reaction mixture was mixed with 1 mL sulfanilic acid reagent (0.33% in 20% glacial acetic acid) and allowed to stand for 5 minutes for the completion of the reaction of diazotization. After this, a further 1 mL of the napthyl ethylene diamine dihydrochloride was added, mixed, and was allowed to stand for 30 minutes at 25°C. The concentration of nitrite was assayed at 546 nm and was calculated with the control absorbance of the standard nitrite solution (without tested sample or standards, but the same conditions should be followed). Here, buffer was used as a blank solution, and Ascorbic acid was taken as a standard solution.

- - 1. *The hydroxyl (OH) radical scavenging assay*

As demonstrated by Kutlu *et al*. (2014), the hydroxyl radical scavenging ability was determined by its capacity to hydroxylate salicylate. The hydroxyl radical was generated by the Fenton reaction between 1.5 mM FeSO4 and 6 mM H_2_O_2_ (1.4:1, v/v) at 37°C for 30 minutes before the assay. 1.0 mL of 1.5 mM FeSO4, 0.7 mL of 6 mM hydrogen peroxide, 0.3 mL of 20 mM sodium salicylate, and varying amounts of the tested samples (0.10, 0.20, and 0.30 mg/mL) were all included in the 3.0 mL reaction mixture. The absorbance of the hydroxylated salicylate complex was measured at 562 nm after a one-hour incubation period at 37°C. The following formula was used to calculate the scavenging activity of the hydroxyl radical effect:

**[1-(A1-A2) / A0] × 100**

Where A0 is the absorbance of the control (without the tested sample) and A1 is the absorbance in the presence of the tested sample, A2 is the absorbance without sodium salicylate.

- - 1. *The hydrogen peroxide (H_2_O_2_) radical scavenging assay*

A solution of hydrogen peroxide (40 mM) was prepared in phosphate buffer (pH 7.4) for the experiment that assessed the effectiveness against hydrogen peroxide (H_2_O_2_) radicals, as recommended by Amessis-Ouchemoukh *et al*. (2017). A hydrogen peroxide solution (0.6 mL, 40 mM) was mixed with 100 *μ*g/mL of each examined component in distilled water. Ten minutes later, the absorbance of hydrogen peroxide at 230 nm was measured in comparison to a blank solution that included phosphate buffer without hydrogen peroxide. The percentages of hydrogen peroxide scavenging for both the tested and reference compounds were calculated.

% Scavenged [H_2_O_2_] = [(AC – AS)/AC] x 100

Where AC is the absorbance of the control, and AS is the absorbance in the presence of the tested sample or standards.

**Table S1.** The *in vitro* antioxidant activity and the scavenging activity of the different synthetic compounds are represented by the inhibition percentages (%) at equal concentrations (10 µg/mL) and the median inhibitory concentrations (IC_50_, µg/mL) against DPPH, ABTS, NO, OH, and H_2_O_2_ radicals.

| **Sample** | | | | **4b** | **4h** | **4i** | **4r** | **4q** | **STD**  **(**Ascorbic Acid**)** | |
| --- | --- | --- | --- | --- | --- | --- | --- | --- | --- | --- |
| **Antioxidant activity** | **TAC**  **(**mg gallic acid/g**)** | | 1 | 52.49 | 17.82 | 38.32 | 66.67 | 83.33 | | 98.75 |
|  |  |  | 2 | 53.75 | 19.25 | 37.24 | 65.27 | 81.23 | | 101.69 |
|  |  |  | 3 | 51.91 | 18.30 | 39.14 | 68.47 | 84.59 | | 97.85 |
|  | **IRP**  **(***µ*g/mL**)** | | 1 | 42.00 | 14.26 | 30.65 | 53.33 | 66.67 | | 79.00 |
|  |  |  | 2 | 43.00 | 15.40 | 29.79 | 52.21 | 64.99 | | 81.35 |
|  |  |  | 3 | 41.53 | 14.64 | 31.31 | 54.78 | 67.67 | | 78.28 |
| **Scavenging activity** | **DPPH** | **Inhibition (**%**)** | 1 | 38.18 | 12.96 | 27.87 | 48.49 | 60.61 | | 71.82 |
|  |  |  | 2 | 39.09 | 14.00 | 27.08 | 47.47 | 59.08 | | 73.95 |
|  |  |  | 3 | 37.76 | 13.31 | 28.46 | 49.80 | 61.52 | | 71.16 |
|  | **ABTS** |  | 1 | 41.93 | 16.71 | 31.62 | 52.24 | 64.36 | | 75.57 |
|  |  |  | 2 | 42.84 | 17.75 | 30.83 | 51.22 | 62.83 | | 77.70 |
|  |  |  | 3 | 41.51 | 17.06 | 32.21 | 53.55 | 65.27 | | 74.91 |
|  | **NO** |  | 1 | 30.43 | 5.21 | 20.12 | 40.74 | 52.86 | | 64.07 |
|  |  |  | 2 | 31.34 | 6.25 | 19.33 | 39.72 | 51.33 | | 66.20 |
|  |  |  | 3 | 30.01 | 5.56 | 20.71 | 42.05 | 53.77 | | 63.41 |
|  | **OH** |  | 1 | 24.34 | 4.17 | 16.09 | 32.59 | 42.29 | | 51.25 |
|  |  |  | 2 | 25.07 | 5.00 | 15.46 | 31.77 | 41.06 | | 52.96 |
|  |  |  | 3 | 24.00 | 4.45 | 16.57 | 33.64 | 43.02 | | 50.73 |
|  | **H_2_O_2_** |  | 1 | 27.26 | 4.67 | 18.02 | 36.50 | 47.36 | | 57.41 |
|  |  |  | 2 | 28.08 | 5.60 | 17.32 | 35.59 | 45.99 | | 59.32 |
|  |  |  | 3 | 26.89 | 4.98 | 18.56 | 37.67 | 48.18 | | 56.82 |
|  | **DPPH** | **IC_50_ (***µ*g/mL**)** | 1 | 9.61 | 28.30 | 13.17 | 7.57 | 6.05 | | 5.11 |
|  |  |  | 2 | 9.97 | 27.84 | 14.39 | 8.21 | 6.60 | | 5.27 |
|  |  |  | 3 | 9.37 | 26.57 | 12.43 | 7.10 | 5.75 | | 4.97 |
|  | **ABTS** |  | 1 | 7.87 | 19.74 | 10.43 | 6.31 | 5.13 | | 4.37 |
|  |  |  | 2 | 8.07 | 19.48 | 11.22 | 6.75 | 5.50 | | 4.45 |
|  |  |  | 3 | 8.39 | 20.42 | 10.81 | 6.51 | 5.34 | | 4.65 |
|  | **NO** |  | 1 | 12.97 | 75.73 | 19.62 | 9.69 | 7.47 | | 6.16 |
|  |  |  | 2 | 13.33 | 66.84 | 21.61 | 10.52 | 8.14 | | 6.31 |
|  |  |  | 3 | 13.85 | 74.71 | 20.06 | 9.88 | 7.73 | | 6.55 |
|  | **OH** |  | 1 | 17.06 | 99.58 | 25.80 | 12.74 | 9.82 | | 8.10 |
|  |  |  | 2 | 17.34 | 86.97 | 28.12 | 13.68 | 10.59 | | 8.21 |
|  |  |  | 3 | 17.52 | 94.52 | 25.38 | 12.50 | 9.78 | | 8.29 |
|  | **H_2_O_2_** |  | 1 | 19.18 | 112.00 | 29.01 | 14.33 | 11.04 | | 9.11 |
|  |  |  | 2 | 19.69 | 98.73 | 31.92 | 15.54 | 12.02 | | 9.32 |
|  |  |  | 3 | 19.93 | 107.51 | 28.87 | 14.22 | 11.12 | | 9.43 |

**Table S2**. The *in vitro* antioxidant activity and the scavenging activity of the different synthetic compounds are represented by the inhibition percentages (%) at equal concentrations (10 *µ*g/mL) and the median inhibitory concentrations (IC_50_, *µ*g/mL) against DPPH, ABTS, NO, OH, and H_2_O_2_ radicals.

| **Sample ID** | | | **4b** | **4h** | **4i** | **4r** | **4q** | **STD**  **(**Ascorbic Acid**)** |
| --- | --- | --- | --- | --- | --- | --- | --- | --- |
| **Antioxidant activity** | **TAC**  **(**mg gallic acid/g**)** | | 52.72±0.54 | 18.46±0.42 | 38.23±0.55 | 66.80±0.93 | 83.05±0.98 | 99.43±1.16 |
|  | **IRP**  **(***µ*g/mL**)** | | 42.18±0.43 | 14.77±0.34 | 30.58±0.44 | 53.44±0.74 | 66.44±0.78 | 79.54±0.93 |
| **Scavenging activity** | **DPPH** | **Inhibition (**%**)** | 38.34±0.39 | 13.42±0.30 | 27.80±0.40 | 48.58±0.67 | 60.40±0.71 | 72.31±0.84 |
|  | **ABTS** |  | 42.09±0.39 | 17.17±0.30 | 31.55±0.40 | 52.33±0.67 | 64.15±0.71 | 76.06±0.84 |
|  | **NO** |  | 30.59±0.39 | 5.67±0.30 | 20.05±0.40 | 40.83±0.67 | 52.65±0.71 | 64.56±0.84 |
|  | **OH** |  | 24.47±0.32 | 4.54±0.24 | 16.04±0.32 | 32.67±0.54 | 42.12±0.57 | 51.65±0.67 |
|  | **H_2_O_2_** |  | 27.41±0.35 | 5.08±0.27 | 17.97±0.36 | 36.59±0.60 | 47.18±0.64 | 57.85±0.75 |
|  | **DPPH** | **IC_50_ (***µ*g/mL**)** | 9.65±0.17 | 27.57±0.52 | 13.33±0.57 | 7.63±0.32 | 6.13±0.25 | 5.12±0.09 |
|  | **ABTS** |  | 8.11±0.15 | 19.88±0.28 | 10.82±0.23 | 6.52±0.13 | 5.32±0.11 | 4.49±0.08 |
|  | **NO** |  | 13.38±0.25 | 72.43±2.81 | 20.43±0.60 | 10.03±0.25 | 7.78 ±0.20 | 6.34±0.11 |
|  | **OH** |  | 17.31±0.14 | 93.69±3.66 | 26.43±0.85 | 12.98±0.36 | 10.06±0.26 | 8.20±0.06 |
|  | **H_2_O_2_** |  | 19.60±0.22 | 106.08±3.90 | 29.93±0.99 | 14.70±0.42 | 11.39±0.31 | 9.29±0.09 |

The values were calculated from n = 3 per sample and are given as mean ± SE.

**Fig. S1**. ^1^H NMR spectrum of compound **4a**.

**Fig. S2**. ^13^C NMR spectrum of compound **4a**.

**Fig. S3**. ^1^H NMR spectrum of compound **4b**.

**Fig. S4**. ^13^C NMR spectrum of compound **4b**.

**Fig. S5**. ^1^H NMR spectrum of compound **4c**.

**Fig. S6**. ^13^C NMR spectrum of compound **4c**.

**Fig. S7**. ^1^H NMR spectrum of compound **4d**.

**Fig. S8**. ^13^C NMR spectrum of compound **4d**.

**Fig. S9**. ^1^H NMR spectrum of compound **4e**.

**Fig. S10**. ^13^C NMR spectrum of compound **4e**.

**Fig. S11**. ^1^H NMR spectrum of compound **4f**.

**Fig. S12**. ^13^C NMR spectrum of compound **4f**.

**Fig. S13**. ^1^H NMR spectrum of compound **4g**.

**Fig. S14**. ^13^C NMR spectrum of compound **4g**.

**Fig. S15**. ^1^H NMR spectrum of compound **4h**.

**Fig. S16**. ^13^C NMR spectrum of compound **4h**.

**Fig. S17**. ^1^H NMR spectrum of compound **4i**.

**Fig. S18**. ^13^C NMR spectrum of compound **4i**.

**Fig. S19**. ^1^H NMR spectrum of compound **4j**.

**Fig. S20**. ^13^C NMR spectrum of compound **4j**.

**Fig. S21**. ^1^H NMR spectrum of compound **4k**.

**Fig. S22**. ^13^C NMR spectrum of compound **4k**.

**Fig. S23**. ^1^H NMR spectrum of compound **4m**.

**Fig. S24**. ^13^C NMR spectrum of compound **4m**.

**Fig. S25**. ^1^H NMR spectrum of compound **4n**.

**Fig. S26**. ^13^C NMR spectrum of compound **4n**.

**Fig. S27**. ^1^H NMR spectrum of compound **4o**.

**Fig. S28**. ^13^C NMR spectrum of compound **4o**.

**Fig. S29**. ^1^H NMR spectrum of compound **4p**.

**Fig. S30**. ^13^C NMR spectrum of compound **4p**.

**Fig. S31**. ^1^H NMR spectrum of compound **4q**.

**Fig. S32**. ^13^C NMR spectrum of compound **4q**.

**Fig. S33**. HPLC purity of compound **4p**.

**Fig. S34**. ^1^H NMR spectrum of compound **4r**.

**Fig. S35**. ^13^C NMR spectrum of compound **4r**.

**Fig. S36**. HPLC purity of compound **4r**.

**References**

Amessis-Ouchemoukh, N.; Ouchemoukh, S.; Meziant, N.; Idiri, Y.; Hernanz, D.; Stinco, C.M.; Rodríguez-Pulido, F.J.; Heredia, F.J.; Madani, K. and Luis, J. (2017). Bioactive metabolites involved in the antioxidant, anticancer and anticalpain activities of *Ficus carica* L., *Ceratonia siliqua* L. and *Quercus ilex* L. extracts. Industrial Crops and Products, 95: 6-17.

Arnao, M.B.; Cano, A. and Acosta, M. (2001). The hydrophilic and lipophilic contribution to total antioxidant activity. Food Chem., 73: 239-244.

Chakraborthy, G.S. (2009). Free radical scavenging activity of *Costus speciosus* leaves. Indian J. Pharm. Educ. Res., 43: 96-98.

Kutlu, T.; Kasim, T.; Bircan, C. and Murat, K. (2014). DNA damage protecting activity and *in vitro* antioxidant potential of the methanol extract of Cherry (*Prunus avium* L). J. Med. Plants Res., 8(19): 715-726.

Oyaizu, M. (1986). Studies on product of browning reaction prepared from glucose amine. Japanese Journal of Nutrition, 44(6): 307-315.

Prieto, P.; Pineda, M. and Aguilar, M. (1999). Spectrophotometric quantitation of antioxidant capacity through the formation of a phosphomolybdenum complex: Specific application to the determination of vitamin E. Anal. Biochem., 269: 337-341.

Rahman, M.M.; Islam, M.B.; Biswas, M. and Alam, A.K. (2015). *In vitro* antioxidant and free radical scavenging activity of different parts of *Tabebuia pallida* growing in Bangladesh. BMC Research Notes, 8(1): 621-628.
